# Design of one-component quasisymmetric protein nanocages

**DOI:** 10.1038/s41586-026-10554-z

**Published:** 2026-05-20

**Authors:** Sangmin Lee, David Chmielewski, Shunzhi Wang, Ryan D. Kibler, Jisu Shin, Ann Carr, Young-Jun Park, David Veesler, David Baker

**Affiliations:** 1 Department of Biochemistry, University of Washington, Seattle, WA, USA.; 2 Institute for Protein Design, University of Washington, Seattle, WA, USA.; 3 Howard Hughes Medical Institute, University of Washington, Seattle, WA, USA.; 4 Department of Chemical Engineering, Pohang University of Science and Technology (POSTECH), Pohang, Republic of Korea.; 5 Institute for Systems Genetics, New York University Langone Medical Center, New York, NY, USA.; 6 These authors contributed equally: Sangmin Lee, David Chmielewski, Shunzhi Wang.

## Abstract

Although the largest completely symmetric closed assembly that can be built from a single building block is the 60-subunit icosahedron^1^, viruses can form capsid assemblies with hundreds to thousands of identical subunits through quasisymmetry—using the same subunit in symmetrically non-equivalent locations in the assembly^2–5^. Quasisymmetric one-component assemblies could have considerable advantages for delivery of biologics because of the large internal volume achieved using only a single building block, but the design of these structures is challenging because of the inherent complexity of designing chemically identical subunits to both adopt different conformations and make different interactions in the distinct symmetrically non-equivalent locations. Here we conjectured that quasisymmetry could arise from spontaneous symmetry breaking in a system of strongly interacting building blocks with programmed curvatures and show that this principle, coupled with a design approach combining a parametric representation of cage architecture with RoseTTAFold diffusion generative modelling, can generate a rich array of quasisymmetric assemblies. Electron microscopy confirmed the structures of designed 3 ≤ *T* ≤ 36 cages with 180–2,160 subunits and diameters from 68 nm to 220 nm, and designed 1 < *T* < 3 non-icosahedral clathrin-like assemblies. Cryogenic electron microscopy structure determination showed how the global symmetry breaking associated with the formation of both hexons and pentons in the *T* = 3 architecture arises from symmetry breaking in the designed subunit interface. Our results indicate how the detailed architecture of complex systems can be controlled by designing overall system properties, and our approach provides a roadmap for designing large quasisymmetric assemblies for biologics delivery and other applications.

Perfect icosahedral assemblies have twenty three-fold symmetry axes that surround twelve five-fold symmetry axes. In many designed and naturally occurring protein icosahedra, three-fold rotational symmetric (C3) protein trimers lie on the three-fold axes such that the 60 subunits of the assembly all have identical chemical environments, with 20 trimers arranged to form 12 pentons^1,6^. To increase the capacity for packaging genetic material without losing economy of genetic encoding, many viruses expand on these structures by inserting additional subunits that form hexagons (hexons) between the pentons (the number of hexons inserted between the pentons depends on the triangulation (*T*) number defined by Caspar and Klug^2^; a regular icosahedron has *T* = 1). Formation of *T* > 1 assemblies requires symmetry breaking (introducing local asymmetry) and multiple subunit conformations, because different locations on the assembly have non-equivalent structural environments (for example, a subunit can border three hexons or two hexons and a penton). In the case of viral capsids, this requires the structural subunit to strike a delicate balance between plasticity (to adopt different conformations in different environments) and precision, as the genetic information must specify a single assembly (or a very restricted set of assemblies) for proper virus function. This balance was optimized in diverse ways over evolutionary time by the strong selective pressure operating on viruses to package maximal genetic material while economizing on the capsid protein(s) coding sequence. In *T* = 3 capsids of diverse animal and plant viruses, chemically identical subunits positioned in distinct local environments of the capsid display conformational switching or disorder-to-order transitions in flexible N-terminal arm domains to stabilize the different subunit–subunit interactions^5–7^. The ability to similarly encode single-component quasisymmetry in designed icosahedral assemblies could enable the generation of larger next-generation synthetic delivery vehicles and protein materials, but how such symmetry breaking can be achieved remains unknown.

Designing quasisymmetric structures is challenging, as identical subunits must adopt distinct conformations and form distinct interfaces at symmetrically non-equivalent sites. We hypothesized that one-component quasisymmetric protein cages with *T* > 1 could be built by globally designing system properties rather than attempting to explicitly optimize the differing interactions made by the subunits in the distinct non-equivalent environments^8,9^. More specifically, we conjectured that symmetry breaking could arise spontaneously in a system of strongly interacting identical subunits tiling a spherically curved surface, driven by the energetic gain of cage closure. Without symmetry breaking, the only possibilities for fully satisfying the bonding potential of the subunits are the flat hexagonal lattice and the perfect icosahedron. Curved spherical surfaces cannot be tiled by hexagonal lattices, and hence to tile surfaces with curvatures lower than that of the regular icosahedron while maintaining full subunit interactions requires interspersing hexagons with pentagons, which, in turn, requires breaking symmetry. We conjectured that the energy gain associated with the formation of additional subunit–subunit interactions on assembly closure could provide a strong driving force for this symmetry breaking, leading to multiple local conformational states (hexamer and pentamer edges) and high *T*-number assemblies. This represents a distinct assembly strategy compared with many native viral capsids, which rely on scaffolding molecules (for example, packaged nucleic acid) within the interior of the capsid^10^. Investigation of native viral capsids, analytically constructed model structures and molecular dynamics simulations (Extended Data Figs. 1–3) suggest that regularly interspersing hexagons with pentagons provides the lowest-energy route to achieving closure while minimizing energetic strain^11,12^. We reasoned further that the triangulation number and size of the resulting assemblies could be controlled through the extent of curvature introduced between adjacent subunits: the smaller the curvature, the higher the *T*-number and size of the resulting assembly.

To explore this design principle, we set out to parameterize the interaction angles between trimeric building blocks spanning the two limiting cases of truly symmetric homogeneous assemblies: *T* = 1 icosahedral cages, consisting entirely of pentons, and two-dimensional (2D) hexagonal lattices, consisting entirely of hexons. *T* > 1 icosahedral cages, consisting of both hexons and pentons, can be regarded as assemblies in between the two boundary structures. As shown in Fig. 1, we define two structure parameters that describe the interaction angle between trimers: *ϕ* and *θ*, where *ϕ* is the angle between vectors **v**_1_ and **v**_2_ from the centre of mass (COM) of a trimer to those of two nearest neighbours (Fig. 1b), and *θ* is the angle between the C3 symmetry axis and the inter-trimer vector **v**_1_ (Fig. 1c) and represents the curvature of the lattice.

The two parameters *ϕ* and *θ* define a 2D parameter space (Fig. 1d), which we explore in detail in this study. The symmetric assemblies at the boundaries (*T* = 1 cages and the 2D hexagonal lattice) are represented by single points because, for each assembly, all the subunits have identical local environments (Fig. 1d–f). If the trimer building blocks are interacting symmetrically, the two parameters are correlated (as *θ* increases, *ϕ* decreases), and lie on the dashed grey line in Fig. 1d. The *T* = 1 cage has *ϕ* = 108° (angle of a regular pentagon) and *θ* ≈ 110°, and the 2D hexagonal lattice has *ϕ* = 120° (angle of a regular hexagon) and *θ* = 90°. We reasoned that between these two reference points would lie parameter sets representing quasisymmetric assemblies. As the local environment of quasisymmetric assemblies is not symmetric (Fig. 1e), a single quasisymmetric cage will map to different points off the correlation line corresponding to the different local geometries generated by the symmetry breaking. For example, the *T* = 3 capsid structure of tomato bushy stunt virus (*T* = 3, PDB id: 2TBV) maps to three different points off the perfect symmetry line (Fig. 1d, cyan points).

To design high-*T*-number quasisymmetric protein cages, we start from homotrimeric protein building blocks (three identical protomers forming a trimeric complex) with arms extending from a central hub and choose the distance *d* between the centres of the building block and the target values of *ϕ* and *θ*. We position three of the C3 building blocks around a central C3 building block at a distance *d* (=|**v**_1_|), and set the angle *θ* by rotating the three flanking building blocks so that their C3 symmetry axes intersect at what will be the centre of the cage (Fig. 1g and Supplementary Fig. 1, rotation 1). The C3 building blocks are rotated again to align the arms of the trimer along the vectors between adjacent building blocks (Supplementary Fig. 1, rotation 2). We then place a C2 building block halfway between two adjacent C3 building blocks with the C2 symmetry axis perpendicular to a vector between the two C3s (Fig. 1h). To fuse the C3 and C2 protomers, we used RFdiffusion^13^ to rigidly connect the two chains (Fig. 1i). The resulting cage subunits, composed of original C3 and C2 protomers fused by the bridging structure, are positioned to interact with each other with the input *ϕ* and *θ* parameters. Because this design strategy uses C3-symmetric oligomers as building blocks, we can only target *ϕ* and *θ* along the correlation line. Our key assumption is that if *ϕ* and *θ* are chosen to have values close to those of a quasisymmetric assembly, symmetry breaking will arise spontaneously to release strain and enable assembly closure, shifting *ϕ* and *θ* off the correlation line^12^.

## Design of symmetric assemblies

**Fig. 2 |:**
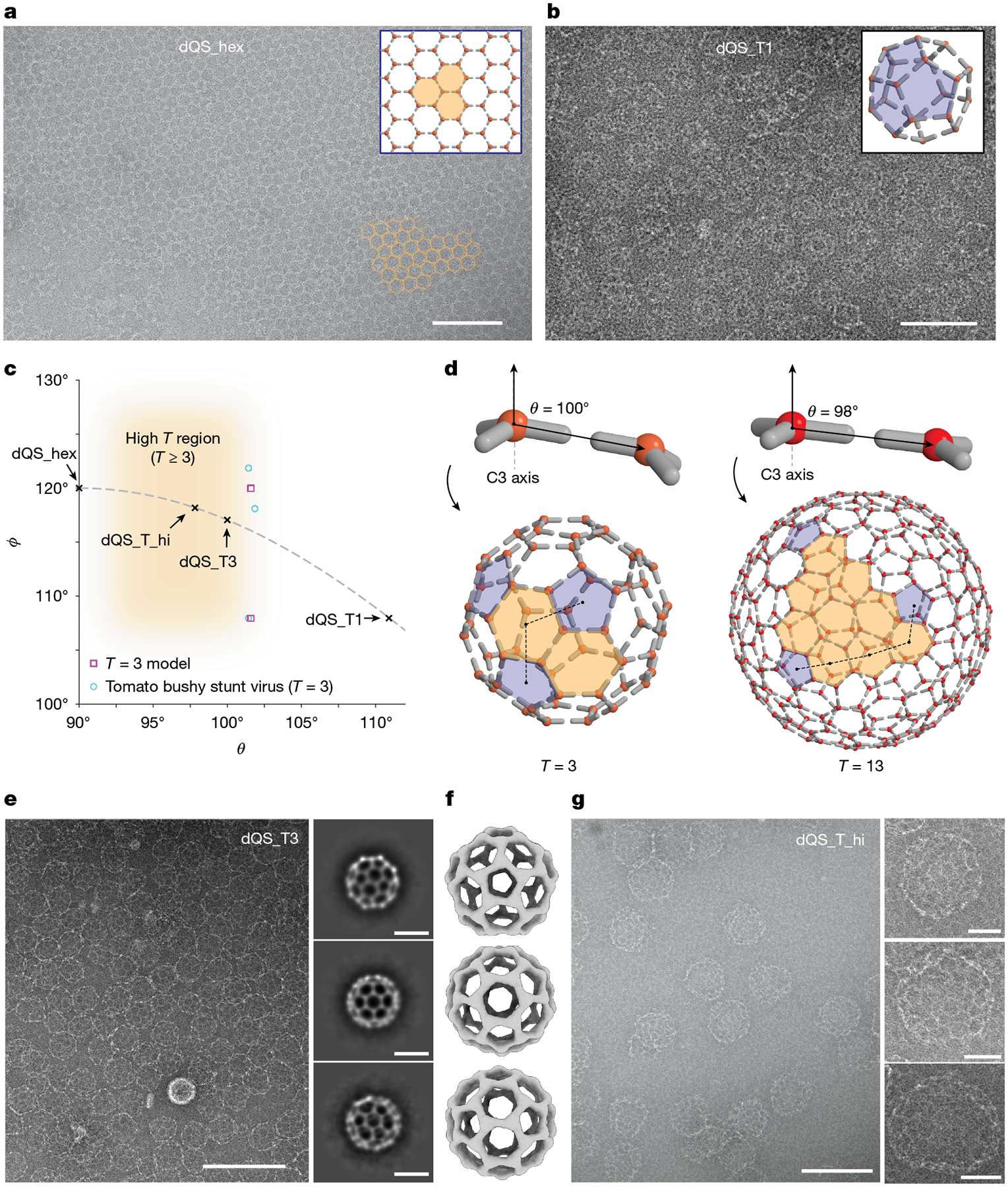
nsEM characterization of high triangulation number assemblies. **a**, Design dQS_hex forms a 2D hexagonal lattice as designed. **b**, Design dQS_T1 forms *T* = 1 cages as designed. **c**, The design parameters (*θ* and *ϕ*) of our experimentally characterized systems span a wide range of values. **d**, The dQS_T_hi assembly has a lower local curvature (top) than the dQS_T3 assembly, resulting in a larger cage (bottom). **e**, nsEM micrograph (left) and 2D averaged classes (right) of dQS_T3. **f**, nsEM 3D reconstructions of dQS_T3 viewed along different rotational symmetry axes. **g**, nsEM micrograph (left) and magnified views (right) of dQS_T_hi cages. Scale bars, 100 nm (**a**,**b**); 200 nm (**e**,**g**); 50 nm (**e**,**g**, insets).

Before exploring the high-*T*-number region, we first designed and tested symmetric assemblies that lie on the boundaries of the design space: the 2D hexagonal lattice (dQS_hex) and the *T* = 1 cage (dQS_T1). We used the design approach described above, first defining the 2D hexagonal lattice by setting *θ* = 90.0 and *ϕ* = 120.0 to form regular hexagons. As building blocks, we used previously validated C3 and C2 designs constructed from repeating helical units^8^. We placed the C3 and C2 units in the targeted geometry with a spacing between COMs of the C3 units of |**v**_1_| = 10.4 nm to provide adequate space to pack two helices in the empty space between each C3 and C2 unit. We then used RFdiffusion^13^ to connect the C3 to C2 unit, providing a previously designed two-helix unit as a guide (Methods). This generated a new protomer backbone consisting of a fusion of the C3 protomer, bridge protein and C2 protomer; the new protomers form new C3 homotrimers that interact with each other with geometry specified by the *ϕ* and *θ* parameters. We designed the amino acid sequence of the new backbone and contacting residues of the C3 and C2 proteins using ProteinMPNN^14^ and selected six designs for which the AlphaFold2 predicted structure was less than 2.5 Å C_α_ root mean square deviation (RMSD) from the computational design model for experimental testing. The selected designs were expressed in *Escherichia coli* and purified by immobilized metal affinity chromatography (IMAC). Two of the six designed proteins were soluble after purification, and one (dQS_hex, Extended Data Table 1) of the two designs was imaged using negative-stain electron microscopy (nsEM), which showed the formation of a 2D hexagonal lattice with an edge length of 10.4 nm (Fig. 2a), close to the design target. We next designed a *T* = 1 symmetric cage with *θ* = 110.9 and *ϕ* = 108.0 corresponding to the angle of a regular pentagon. We expressed six of the designed proteins in *E. coli* and purified them by IMAC. Five out of the six designs were soluble after purification, and one (dQS_T1; Extended Data Table 1) of the five designs was characterized by nsEM. The nsEM imaging showed homogeneous populations of *T* = 1 cages resembling the design models (Fig. 2b and Supplementary Figs. 8 and 9). Thus, our design approach can robustly generate symmetric assemblies.

## Design of high-*T*-number assemblies

**Extended Data Fig. 1 |:**
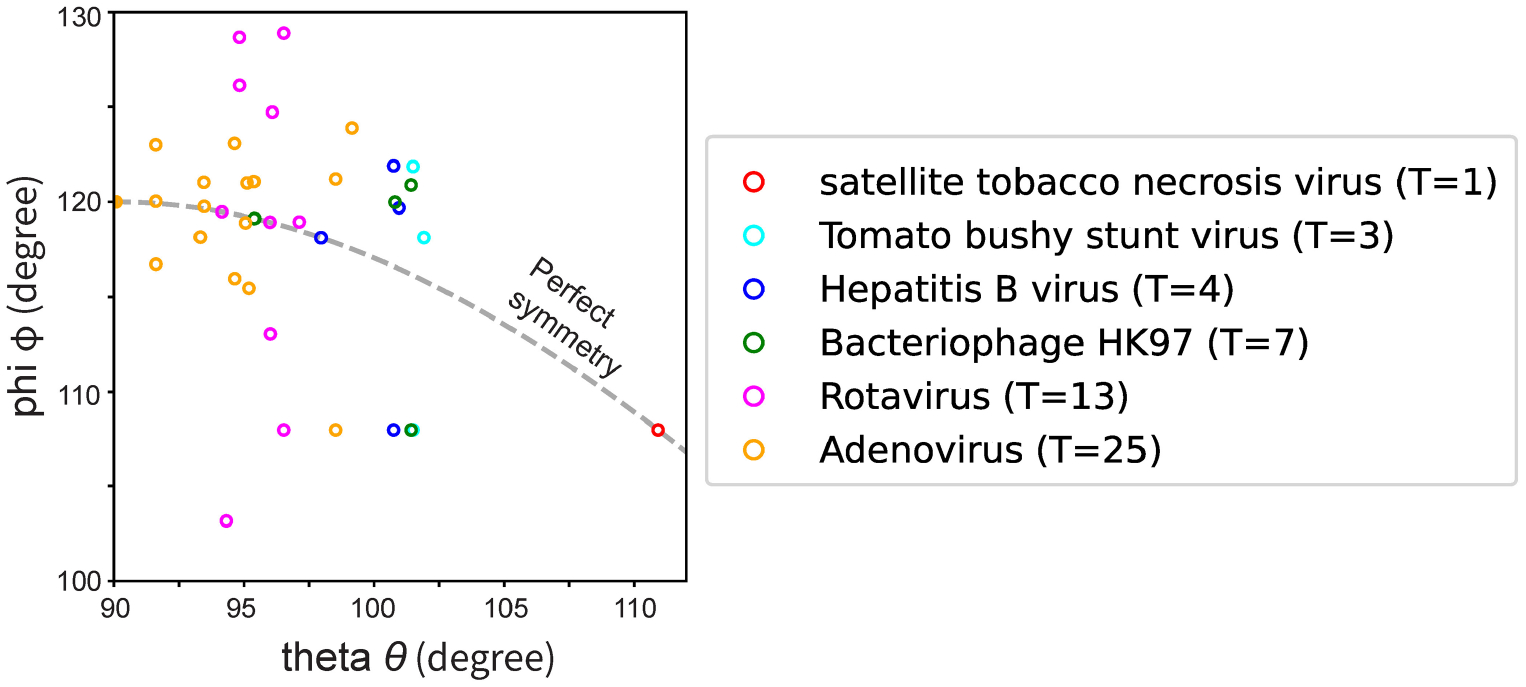
Real viral capsids mapped to the design space. Satellite tobacco necrosis virus (PDB: 2BUK). Tomato Bushy stunt virus (PDB: 2TBV). Hepatitis B virus (PDB: 1QGT). Bacteriophage HK97 (PDB: 1OHG). Rotavirus (PDB: 3KZ4). Adenovirus (PDB: 6CGV). The design parameters (*θ* and *ϕ*) are defined in Fig. 1. The scattered points out of the perfect symmetry line suggest that the symmetry of capsid proteins is broken by forming the closed architectures.

**Extended Data Fig. 2 |:**
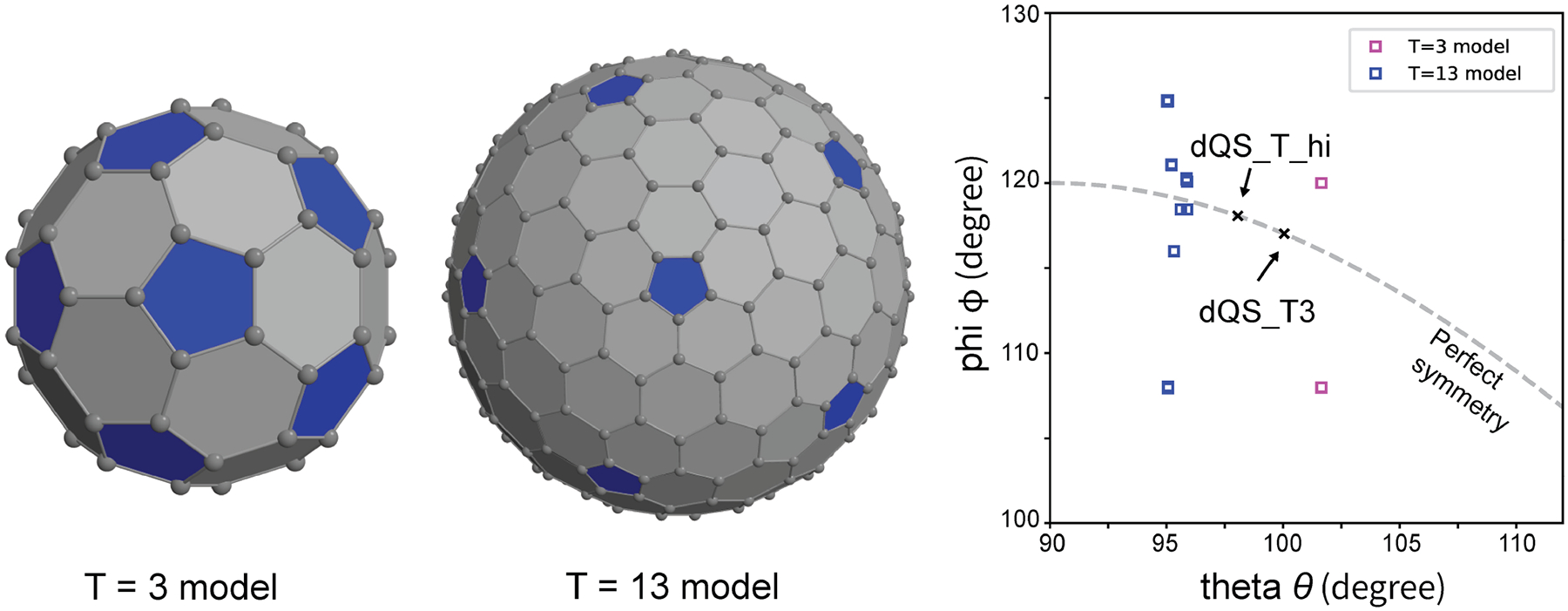
Analytical constructed model structure of T = 3 and T = 13 cages. (left) Model structure of T = 3 and T = 13 cages. (right) Structure parameters (*θ* and *ϕ*) of the two model structures. The mapped parameters (data points in the plot) of the T = 13 model are more scattered than those of the T = 3 model, indicating that more unique local environments are formed for higher T-number cages.

**Extended Data Fig. 3 |:**
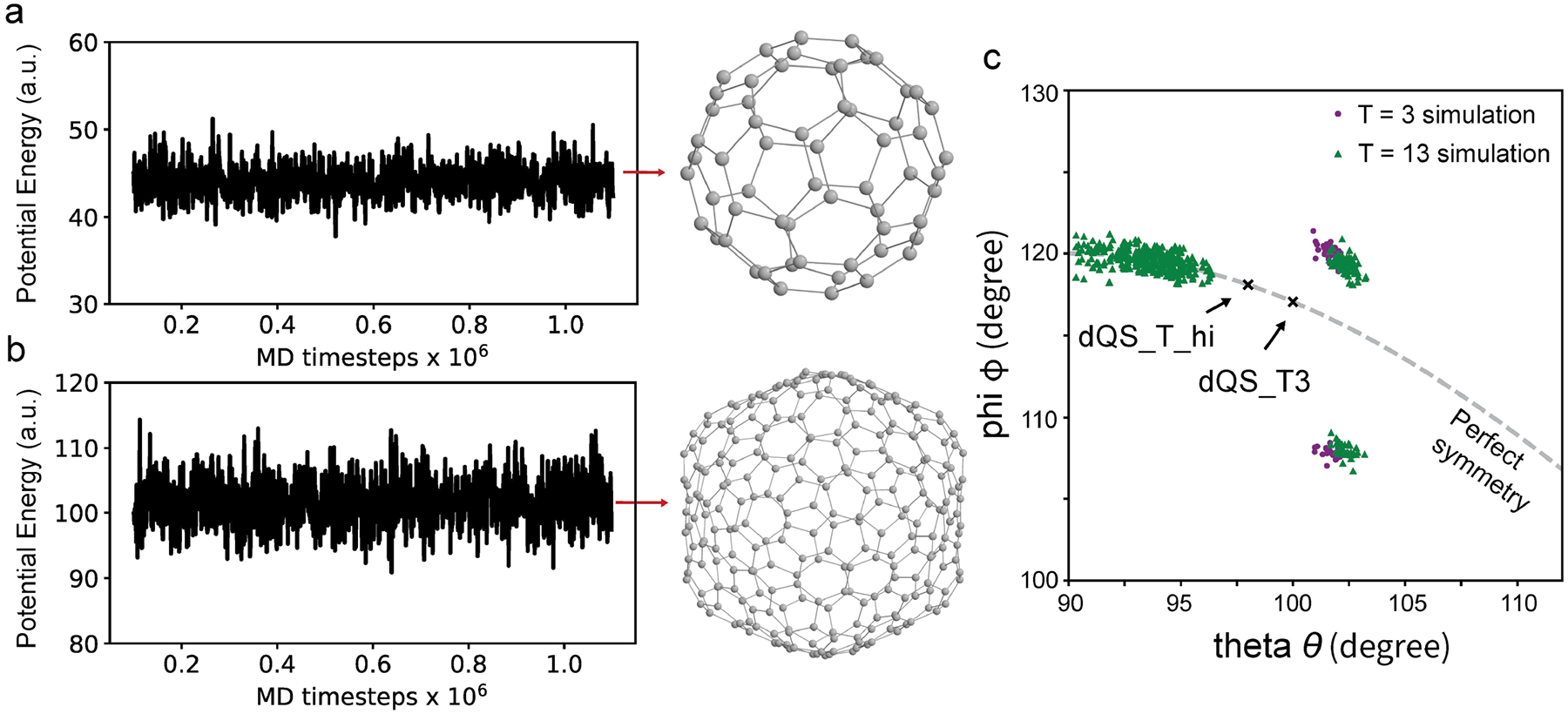
MD simulations for thermally equilibrated T = 3 and T = 13 cage structures. (**a**,**b**) Time evolution of potential energy during equilibration of the cages for 10^6^ MD timesteps. Fluctuation along a constant value indicates that the systems are equilibrated. (**c**) *θ* and *ϕ* of each cage obtained from the equilibrated structures.

Next, we aimed to design high-*T*-number (*T* > 1) quasisymmetric cages. From analysis of both simulated structures and high-*T*-number icosahedral viral capsids within the *ϕ* and *θ* parameter space (Extended Data Figs. 1–3), we found that *T* ≥ 3 cages have *θ* between 90° and 102° (Fig. 2c). We began by targeting *T* = 3 cages for which we derived values of *θ* = 100.0° and *ϕ* = 117.1° from an analytically constructed model structure (Fig. 2d and Extended Data Fig. 2). Similar to the design approach used above for the symmetric assemblies, we first placed the pre-validated C3 and C2 units (|**v**_1_| = 12.5 nm) according to the targeted parameters and then connected the two units with a four-helix bundle using RFdiffusion. We reasoned that a combination of local rigidity and global deformability could be optimal for achieving the desired symmetry breaking and used building blocks with interactions primarily between residues close along the sequence (low contact order) and relatively small subunit–subunit interfaces.

We selected six *T* = 3 designs for experimental characterization, expressed the proteins in *E. coli*, and purified them by IMAC. Five out of six of the designed proteins were soluble after purification, and three out of six showed a sharp peak on size-exclusion chromatography (SEC) at retention volumes consistent with the design models (Supplementary Fig. 4). One of the three designs that shows the most homogenous peak in the SEC traces and dynamic light scattering (Supplementary Figs. 9 and 10) was characterized by negative-stain electron microscopy (nsEM). Homogeneous populations of about 70 nm size cages were observed in nsEM micrographs, whereas 2D class averages (Fig. 2e) and a three-dimensional (3D) reconstruction showed an overall structure very close to the design model structure (Fig. 2f and Methods). The *T* = 3 icosahedral cage (dQS_T3) (Extended Data Table 1 and Supplementary Fig. 3) has a soccer ball-like structure with 12 pentagons aligned along the five-fold symmetry axes, with each pentagon surrounded by five hexagons that are each aligned along three-fold symmetry axes, resulting in an assembly with 180 subunits in total. The EM structural data confirm the quasisymmetry of the assembly, with subunits adopting different conformations to form pentons and hexons.

To explore the generation of larger higher-*T*-number cages, we studied the lower *θ* region of the parameter space in which natural viral capsids with higher *T*-numbers are located (Extended Data Fig. 1). We tested a system with *θ* = 98.0° and *ϕ* = 118.1° (dQS_T_hi, Fig. 2c), similarly constructed from a designed homotrimer and C2 interface^8^. The designed proteins were again expressed in *E. coli* (Supplementary Fig. 3), and their assembled structures were characterized by nsEM after IMAC and SEC purification. Consistent with our design concept that reducing curvature should increase assembly size, the cages observed in the nsEM micrographs are much larger (diameter >120 nm; Supplementary Fig. 9) than the dQS_T3 cages (approximately 70 nm diameter) although the sizes and shapes are less homogeneous (Fig. 2g). Cryo-electron microscopy (cryo-EM) micrographs (Fig. 4 and Supplementary Fig. 2) confirmed cages with diameters ranging from 120 nm to 220 nm, which corresponds to 13 ≤ *T* ≤ 36 (number of subunits: 780–2,160) based on the ratio between the edge length and the diameter of the cages (Supplementary Fig. 5). Heterogeneity is not surprising in high-*T*-number cage systems because the parameter differences (*θ* and *ϕ*) between neighbouring *T*-number cages become smaller as the *T*-number increases. Unlike native viral capsids, assembly of the designed nanocages is driven solely by the interaction between subunits; borrowing from the example of natural high-*T*-number viral capsids^15–17^, achieving a more homogenous cage size in designed high-*T*-number systems could be accomplished by using size-regulating scaffolding proteins or nucleic acids in the interior, or spherical assemblies (proteins, lipids and polymers) with defined sizes to direct lattice assembly.

## Cryo-EM analysis of symmetry breaking

**Fig. 3 |:**
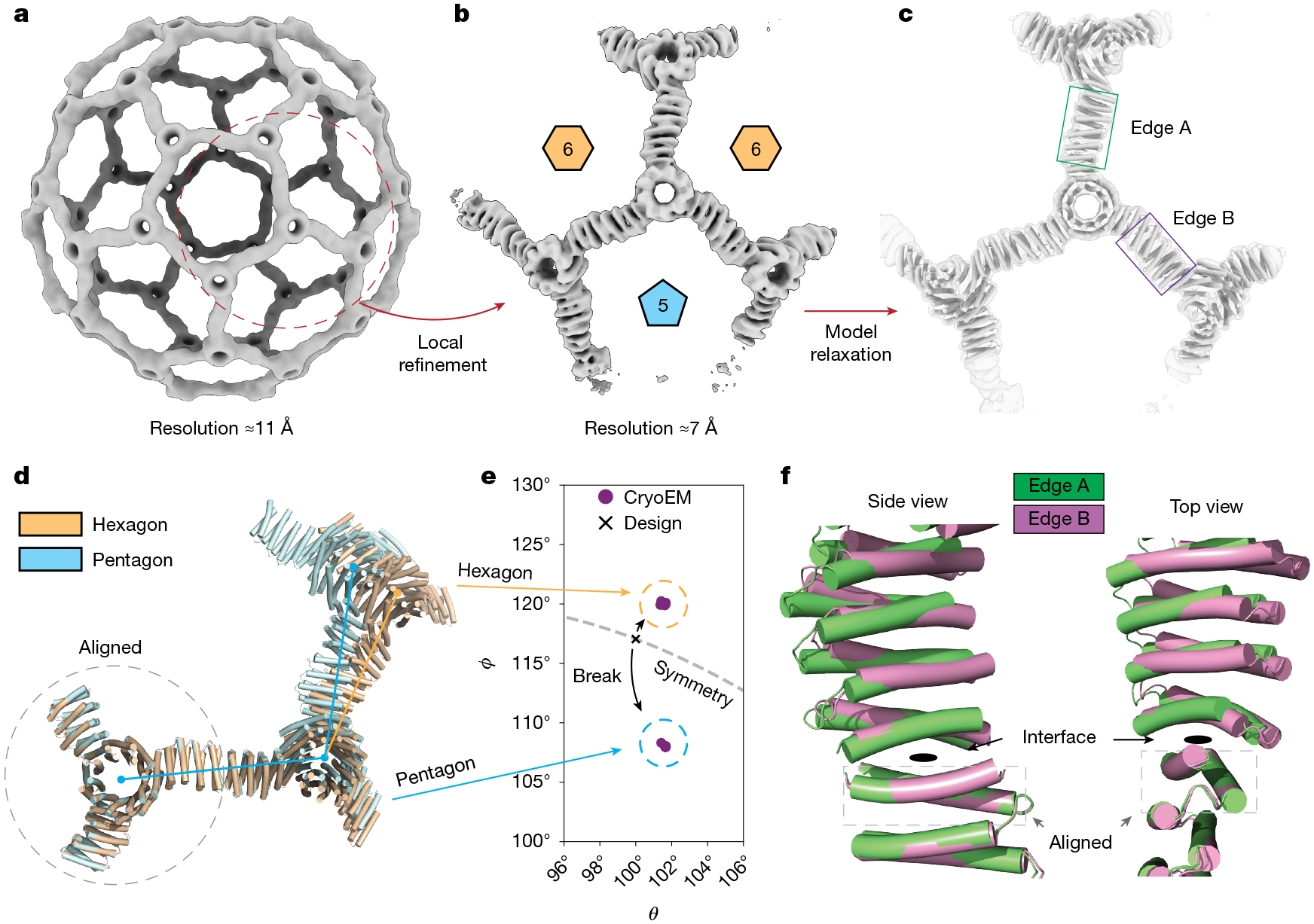
Cryo-EM analysis of symmetry breaking in the dQS_T3 structure. **a**, Cryo-EM 3D reconstruction of dQS_T3 cage. **b**, Local focused refinement increases the resolution to 7 Å. **c**, Relaxation of protein model into the cryo-EM map. **d**, Comparison of relaxed models between the three trimers forming a pentagon and the three trimers forming a hexagon. **e**, The *θ* and *ϕ* values calculated from the model structure deviate from perfect symmetry (grey dotted line), as intended by design. **f**, Interface between two hexagons (green). C2 interface between pentagon and hexagon (purple).

To understand the symmetry breaking in the subunits of the *T* = 3 cage in more detail, we collected cryo-EM micrographs of purified dQS_T3 cages (Extended Data Table 2) and determined a reconstruction at about 11.0 Å resolution applying global icosahedral symmetry over the multiple-subunit extended asymmetric unit containing one penton and the adjacent trimers (Fig. 3a). We observed polymorphism between individual cages, and to increase resolution of self-consistent sub-particle regions (Methods) we carried out local focused refinement^18^ over focus regions consisting of four neighbouring trimers, which encompass the two distinct local environments: the edge between two hexagons (edge A) and the edge between pentagon and hexagon (edge B) (Fig. 3b). The focused classification and refinement increased resolution to about 7.0 Å, enabling the identification of helical secondary structure features (Fig. 3b). We carried out density-guided model relaxation by ISOLDE^19^, which places helices of the model into the density (Fig. 3c). In the relaxed model, distinct local structures form the hexagons and the pentagons (Fig. 3d), which map to two different clusters with different *ϕ* values (about 108° and 120°) in the design parameter space (Fig. 3e). The two clusters are located off the symmetry correlation line, consistent with local symmetry breaking and the overall design concept. To understand the structural origin of the symmetry breaking, we aligned the relaxed model structures of the two different edges (edges A and B) (Fig. 3b) and found that the deviations were primarily at the subunit interfaces (Fig. 3f). Together with a previous report of quasisymmetric cages driven by local structural flexibility^20^, our results indicate that quasisymmetry can be driven by deformation of either the subunit interfaces or the subunit backbone, or by a combination of both.

**Fig. 4 |:**
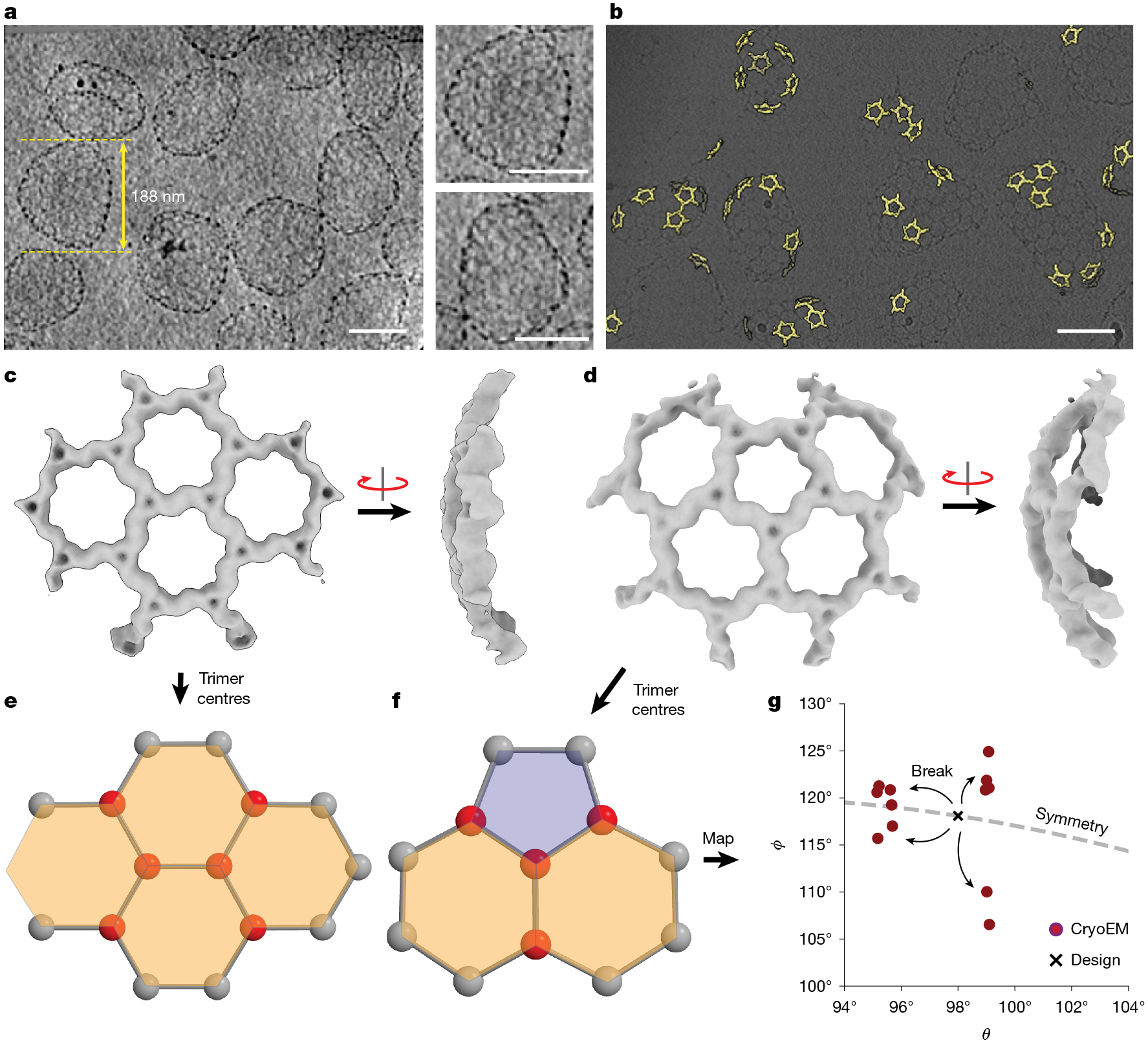
Symmetry breaking in the high triangulation number dQS_T_hi assembly. **a**, Cryo-ET images showing diverse morphologies of dQS_T_hi particles. **b**, Mapping refined pentagonal motifs to an original tomogram image. **c**,**d**, Three-dimensional reconstructions of regions consisting of only hexagons (**c**) and both pentagons and hexagons (**d**). **e**,**f**, Ball-and-stick models constructed from the subvolume maps shown in **c** and **d**. Each bead represents the centre of a trimer, and the *θ* and *ϕ* of red beads are shown in the design parameter space in **g**. **g**, The *θ* and *ϕ* values calculated from the subvolume maps deviate from the perfect symmetry line (grey dotted line), and occupy a wider range of values than in the case of the dQS_T3 design in Fig. 3. Scale bars, 100 nm (**a**,**b**); 100 nm (**a**, insets).

The symmetry breaking of higher-*T*-number cages (dQS_T_hi, *T* ≥ 13) occurs in even more complex ways. We observed heterogeneity of the cages in both diameter and shape (Fig. 4a). The observed diameters range from 120 nm to 220 nm (Supplementary Fig. 2), and the overall shape of the cages ranges from spherical to HIV-capsid-like (fullerene cone) structures^21,22^. Owing to the marked heterogeneity, we conducted cryogenic electron tomography (cryo-ET) and performed subvolume analysis to reconstruct 3D tomogram volumes of sub-regions of the cages (Fig. 4b–d and Extended Data Table 3). We determined the locations in full assembly tomograms of refined pentons generated from this sub-region analysis (Fig. 4b). We found that pentons on each cage were not consistently positioned with respect to each other (consistent with the heterogeneous cage shapes), with different organizations in different cages. We obtained cryo-ET maps of two representative sub-regions that highlight the quasisymmetric characters of the cages: a sub-region in which only hexagons are presented (Fig. 4c) and another sub-region in which a pentagon is surrounded by hexagons (Fig. 4d). We transformed the cryo-ET subvolumes to ball-and-stick models (Fig. 4e,f), in which each sphere represents the centre of trimers, to map the subvolumes to the parameter space and identify the local symmetry breaking (Fig. 4e). The *ϕ* and *θ* values obtained from the cryo-ET maps deviate more from the symmetry correlation line (Fig. 4g) than the *T* = 3 cages (Fig. 3e); higher-*T*-number cages contain more diverse local environments that break symmetry in different ways.

## Design of sub-*T* = 3 region and boundaries

**Extended Data Fig. 4 |:**
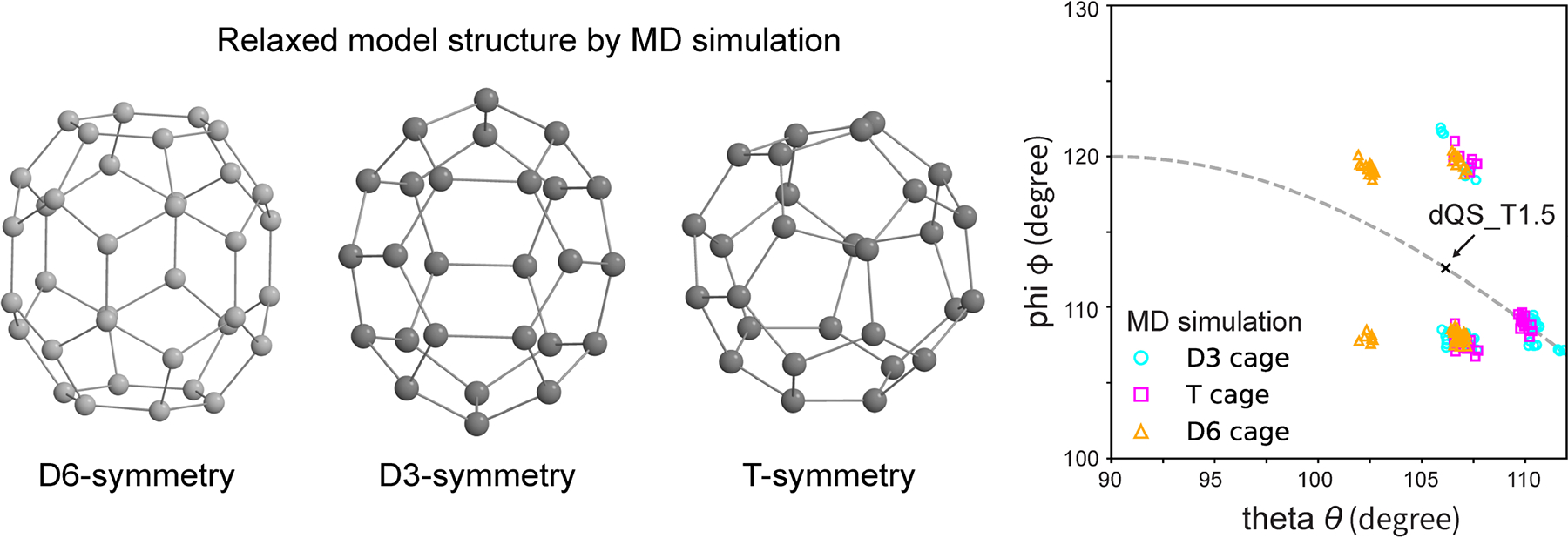
MD simulations for thermally equilibrated sub-T3 cages (D6, D3, T symmetries). (left) Relaxed cages by MD simulations for 10^6^ MD timesteps. (right) Clusters of *θ* and *ϕ* of each cage mapped to the parameter space.

**Fig. 5 |:**
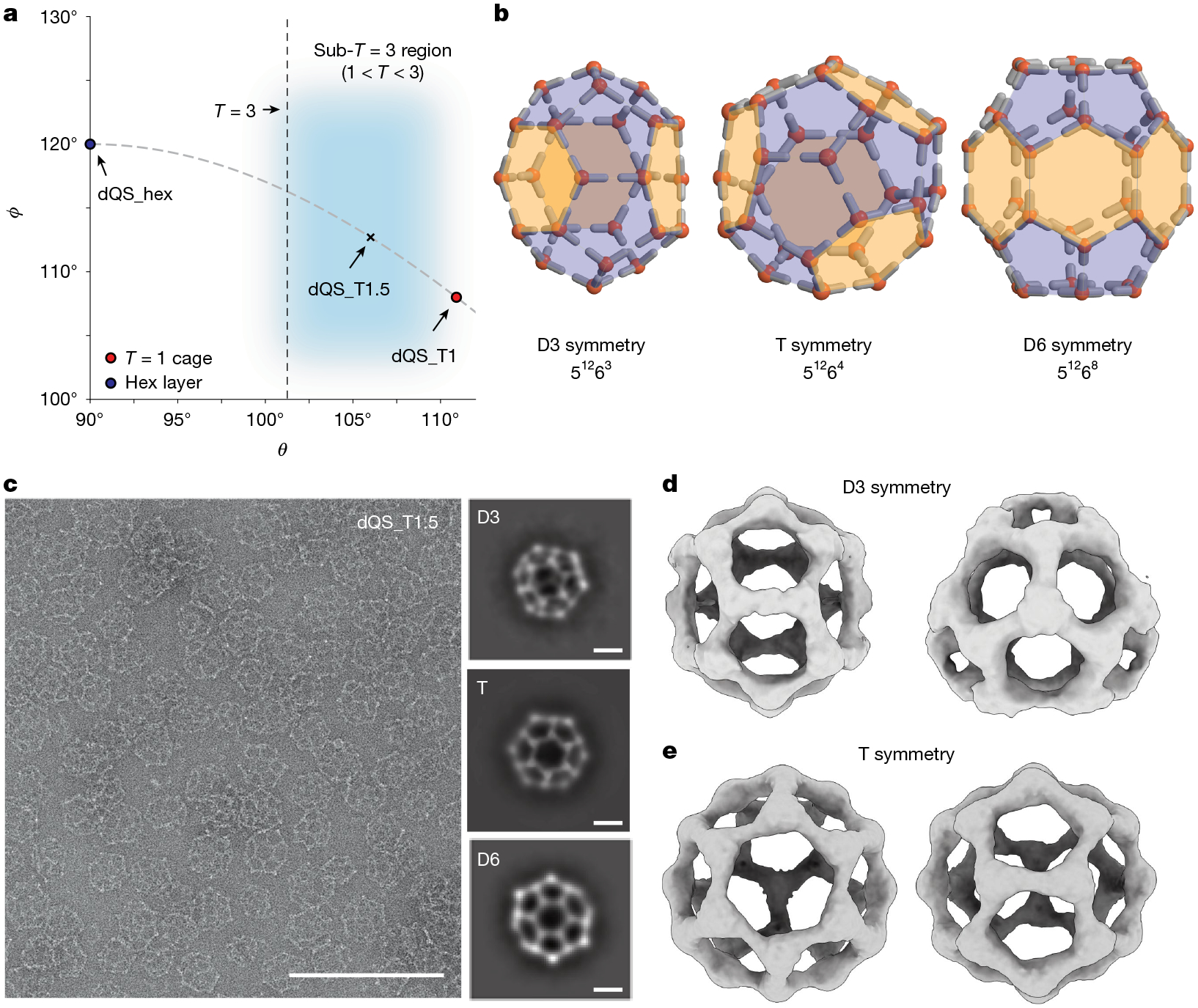
Quasisymmetric design space between *T* = 1 and *T* = 3. **a**, Design parameters (*θ* and *ϕ*) of dQS_hex, dQS_T1.5 and dQS_T1. **b**, Model structures of non-icosahedral quasisymmetric cages. **c**, nsEM micrograph of dQS_T1.5 (left) and 2D averaged classes showing a mixture of multiple non-icosahedral sub-*T* = 3 quasisymmetric cages (right). **d**,**e**, nsEM 3D reconstructions of D3 and T symmetric cages of dQS_T1.5. Scale bars, 200 nm (**c**); 20 nm (**c**, insets).

According to the standard definition of *T*-number^2^, there are no architectures with 1 < *T* < 3, but there is a large region between *T* = 1 and *T* = 3 in our design space (Fig. 5a), which we refer to as the sub-*T* = 3 region. Analysis of simulated model structures (Extended Data Fig. 4) showed that non-icosahedral quasisymmetric D3, D6 and tetrahedral (T) symmetric cages can be generated in the sub-*T* = 3 region (Fig. 5b). Similar to the icosahedral high-*T*-number cages, the non-icosahedral cages consist of 12 pentagons and several hexagons, and hence these cages can be viewed as being between *T* = 1 and *T* = 3 cages. With the nomenclature of 5^*n*^6^*m*^, where *n* and *m* are the numbers of pentagons and hexagons, respectively, the icosahedral *T* = 1 and *T* = 3 cages are 5^12^ and 5^12^6^12^, and the sub-*T* = 3 non-icosahedral cages can be written as 5^12^6^3^ (D3 symmetry), 5^12^6^4^ (T symmetry) and 5^12^6^8^ (D6 symmetry). These cage structures are often found in gas hydrates^23^ and colloidal crystals^24^ and are known as Frank–Kasper polyhedra^25^; in biology, these structures are best represented by clathrin structures^26^.

We designed and experimentally tested a system (dQS_T1.5) with *θ* = 106.0° and *ϕ* = 112.7° in the sub-*T* = 3 region. The sub-*T* = 3 cages obtained by *E. coli* expression were characterized by nsEM, which revealed a mixture of several types of cage structure (Fig. 5c and Extended Data Table 1). This mixture of sub-*T* = 3 architectures is often observed in clathrin systems^26^, which have local structures similar to our designed cages. The 2D class averages are consistent with D3 symmetry (78 subunits), D6 symmetry (108 subunits) and T symmetry (84 subunits) cages with diameters of 55.6 nm, 65.1 nm and 59.8 nm, respectively (Fig. 5c). Three-dimensional reconstructions of the D3 symmetry and T symmetry cages indicated non-icosahedral quasisymmetric architectures composed of pentons and hexons (Fig. 5d,e), which match well with the model structures (Fig. 5b).

## Conclusion

Our results support our conjectures that quasisymmetry can arise from spontaneous symmetry breaking in a system of strongly interacting building blocks, driven by the energetic gain from assembly closure, and that the triangulation number of the resulting assembly can be controlled by the curvature of the interacting building blocks. We show that these principles enable the design of well-defined *T* = 3 cages, less homogeneous *T* ≥ 13 cages and Frank–Kasper polyhedra. Whereas almost all previous computational design of protein nanomaterials has sought to precisely specify the atomic interactions between building blocks, here we instead focus on designing overall system properties. The accompanying paper^27^ demonstrates the generality of this design principle through the construction of two-component quasisymmetric structures. We suspect that as biological design efforts move up the complexity ladder, these design principles and approaches will become increasingly important.

We speculate that the evolution of *T* > 1 viral capsids likewise took advantage of spontaneous symmetry breaking arising from mutations that altered the overall curvature of interacting subunits, rather than mutations that directly stabilized the multiple distinct interfaces. To increase the homogeneity of the higher triangulation number assemblies once populated, these assemblies were probably stabilized through the introduction of additional scaffolding and host factor interactions, and optimization of the different subunit–subunit interfaces. Likewise, to reduce the heterogeneity of our higher-*T*-number assemblies, protein design could follow natural evolution here and use analogous interacting and scaffolding components to stabilize specific target quasisymmetric assembly architectures.

## Methods

### Computational methods

#### Computational design protocol of single-component high-*T*-number cages.

We first constructed input architectures for a given set of parameters (*d*, *θ* and *ϕ*) using (1) C3 oligomers; (2) bridge proteins; and (3) C2 interfaces. After the construction, three parts (a C3 protomer, a bridge protein and a C2 protomer) were connected in terms of indices of atoms and amino acids (that is, we merged the three chains as one chain in *.pdb file format). The merged chain was used as an input of RFdiffusion, and the bridge protein was partially diffused (diffuser. partial_T = 20) to rigidly connect the three parts together. The outputs of RFdiffusion^13^ were used as inputs of proteinMPNN^14^ to design a library of amino acid sequences (diffusion temperature = 0.1, 0.15, 0.2, 0.25, 0.3, 0.4, 0.5; 32 sequences for each temperature), which are predicted to stabilize the generated backbone. Folded structure of each sequence of the library was predicted by AlphaFold-2 (ref. 28), and C_α_ RMSD was calculated between the predicted structure and the generated backbone (= the target structure we want) (Supplementary Fig. 6). Designed sequences with the RMSD less than 2.5 Å were used for experimental tests. More details of the initial model construction are described below.

#### Model construction.

We first defined the COMs (COM1, COM2, COM3 and COM4) of C3 oligomers for the given parameters. The **v**_1_ was defined as a vector from COM1 to COM2, and **v**_2_ was from COM1 to COM3. Then, we read the *xyz* coordinates of the C3 oligomer, duplicated the coordinates into four (that is, four identical C3 oligomers) and translated each oligomer to each COM. Then, each oligomer was rotated to align the C3 rotational axis with a vector from the origin (= 0, 0, 0) to each COM. Then, each oligomer was rotated again to align the direction of an arm of the oligomer along a vector from the COM of the oligomer to the COM of a neighbouring oligomer (Supplementary Fig. 1). Next, we read the *xyz* coordinates of the C2 oligomer, duplicated the coordinates into three, and translated each C2 oligomer to the centre of COM1–COM2, COM1–COM3 and COM1–COM4. Then, each C2 oligomer was rotated to align the rotational symmetry axis of the C2 with a vector from the origin to each COM of C2. Then, a bridge protein consisting of four alpha helices was roughly placed (we used ChimeraX to drag it to an appropriate place) between the C3 and C2 oligomers. Finally, the three parts (C3 protomer, C2 protomer and bridge protein) were merged as one chain in a *.pdb file format, which was used as an input of RFdiffusion in the next step. We first constructed the initial configuration using four C3 oligomers to check the overall structure, but at the end, only one set of the three parts (a C3 protomer, a bridge protein and a C2 protomer) was needed for the next steps, because all the construction was performed symmetrically. We have attached a sample code of the model construction and input files.

#### Molecular dynamics simulation.

Simulations were conducted using molecular dynamics (MD) implemented in the HOOMD-blue particle simulation package (https://github.com/glotzerlab/hoomd-blue)^29^. In the simulation, the model cages are represented as vertices of pentagons and hexagons of the cage surface (see the model structure of cages in Extended Data Figs. 2 and 3), and the vertices are represented as spherical beads (diameter 0.3*σ*). Each bead is bonded with the nearest neighbouring beads by a harmonic bond potential (applied to pairs forming a bond), $V_{\text{bond}}=\frac{1}{2}k_{\text{bond}}{\left(r-r_{0}\right)}^{2}$, where *k*_bond_= 100*ε*/*σ*^2^, *r*_0_= 1.1*σ*, and by an angular potential (applied to triplets forming a bond angle), $V_{\text{angle}}=\frac{1}{2}k_{\text{angle}}{\left(\theta_{h}-\theta_{h\_0}\right)}^{2}$, where *θ*_0_ = 120° and *k*_angle_ = 20*ε*. Here, *r* is the distance between the centres of beads, and *θ*_h_ is the bond angle of the triplet; *ε* is the energy unit, and *σ* is the length unit of the simulation.

The constructed model cages were used as initial configurations and first thermalized for 10^5^ MD timesteps (with d*t* = 10^−4^*τ*, where *τ* is the simulation time unit) under Brownian dynamics. Then, the systems were equilibrated for 10^6^ MD timesteps by monitoring potential energy (until it reaches a plateau) at a constant temperature (T* = 0.2*kT*/*ε*). After equilibration, the positions of the cage vertices were time-averaged for 5 × 10^4^ MD timesteps to minimize noises, and the design parameters (*θ* and *ϕ*) were calculated.

#### Calculating potential energy landscape across *T*-numbers.

In the simulation model, the interactions between nodes of the cages are described by two types of potential energies: (1) a harmonic bond potential for stabilizing bond distances and (2) a harmonic angle potential for stabilizing bond angles. To calculate potential energy landscapes, we first defined two sets of potential energy parameters, for which the potential energy is minimized for ideally constructed *T* = 3 (*θ* = 101° and *ϕ* = 116°) and *T* = 13 (*θ* = 95° and *ϕ* = 119°) models, respectively. We then applied each parameter set to different *T*-number cage models (*T* = 3, 9, 13, 16 and 36) to calculate the potential energy landscape across *T*-numbers for each parameter set.

We proposed that if the potential energy minimum for a target *T*-number cage is distinctly lower than those of other *T*-number cages, higher structural homogeneity would be observed at equilibrium (that is, only the target *T*-number cages would form). By contrast, if the energy differences are small, structural diversity would emerge (that is, several *T*-number cages would be populated).

Simulation results show that, for the parameter targeting *T* = 3, the potential energy increases rapidly as the *T*-number deviates from the target value. By contrast, for the parameters targeting *T* = 13, the potential energies of *T* = 13 and its neighbouring *T*-numbers (*T* = 9 and *T* = 16) are comparable (Supplementary Fig. 7).

These results indicate that, for high-*T* cages, the energy differences between adjacent *T*-numbers are not sufficiently distinct, leading to the observed structural diversity in this study. This further implies that an additional size-defining scaffold (for example, RNA or DNA in viruses) may be required to achieve structural homogeneity in high-*T* cages^12^.

### Experimental methods

The experimental methods and materials used in this study are mostly the same methods and materials as described in our previous studies^8,30^.

#### Protein expression. Protocol.

Synthetic plasmids (100 ng) were transformed into a chemically competent *E. coli* expression strain, BL21(DE3), using 10 μl competent cells per reaction, following the protocol of the manufacturer. Following transformation and recovery, the entire transformation products were used to inoculate 1 ml Luria–Bertani medium containing 100 μg ml^−1^ kanamycin and grown at 37 °C with shaking at 200 rpm overnight. Of overnight cultures, 500 μl was diluted into 50 ml TBM-5052 supplemented with 100 μg ml^−1^ kanamycin in 250 ml baffled flasks and incubated at 37 °C with shaking at 200 rpm for 18–24 h.

#### Buffers.

All buffers and media were made using Milli-Q filtered water.

Autoinduction medium (TBM-5052): 1.2% (wt vol^−1^) tryptone, 2.4% (wt vol^−1^) yeast extract, 0.5% (wt vol^−1^) glycerol, 0.05% (wt vol^−1^) d-glucose, 0.2% (wt vol^−1^) d-lactose, 25 mM Na_2_HPO_4_, 25 mM KH_2_PO_4_, 50 mM NH_4_Cl, 5 mM Na_2_SO_4_, 2 mM MgSO_4_, 10 μM FeCl_3_, 4 μM CaCl_2_, 2 μM MnCl_2_, 2 μM ZnSO_4_, 400 nM CoCl_2_, 400 nM NiCl_2_, 400 nM CuCl_2_, 400 nM Na_2_MoO_4_, 400 nM Na_2_SeO_3_ and 400 nM H_3_BO_3_.

Lysis buffer: 25 mM Tris, 300 mM NaCl, 20 mM imidazole, 10% glycerol and pH 8.0 at room temperature.

Wash buffer: 25 mM Tris, 300 mM NaCl, 40 mM imidazole, 10% glycerol and pH 8.0 at room temperature.

Elution buffer: 25 mM Tris, 300 mM NaCl, 300 mM imidazole, 100 mM EDTA, 10% glycerol and pH 8.0 at room temperature.

SEC buffer: 25 mM Tris, 300 mM NaCl and pH 8.0 at room temperature.

#### Protein purification. Immobilized metal affinity chromatography.

Cultures were harvested by centrifugation at 4,000*g* for 15 min, the culture supernatant was decanted, and pellets were resuspended in 35 ml of lysis buffer. For cell lysis, 300 μl of PMSF (100 mM in 100% ethanol) was added immediately before sonication at 70% power for 5 min. Then, lysates were clarified by ultracentrifugation at 18,000*g* at 12 °C for at least 30 min and applied to 1.5-ml of Ni-NTA resin (Qiagen) pre-equilibrated with lysis buffer and packed into Econo-Pac columns (Bio-Rad) for gravity chromatography. The columns were washed twice with 15 ml of wash buffer and eluted with 10 ml of elution buffer. Hetero-oligomers were purified according to a similar procedure, except they used the ‘Het’ variants of the lysis, wash and elution buffers, as this was found to improve the yield of single-His-tagged complexes over nonspecific multiple-His-tagged complexes, which can arise at high concentrations and probably dominate binding to the Ni-NTA resin. Samples prepared for crystallization were treated similarly, except that 500-ml cultures were used and the lysate was divided among six gravity columns.

#### Size-exclusion chromatography.

IMAC-eluted samples were concentrated using 10k MWCO spin concentrators and were purified using a Superdex 6 10/300 increase column (Cytiva) in SEC buffer using an ÄKTA pure system (Cytiva). SEC traces were also used to qualitatively determine homogeneity and quantitatively measure total yield by A280 absorbance integrated over the collected fractions using Unicorn (Cytiva).

#### Sample analysis. Liquid chromatography mass spectrometry.

To identify the molecular mass of each protein and thus verify sample identity and integrity, intact mass spectra were obtained using reverse-phase liquid chromatography mass spectrometry on an Agilent G6230B TOF on an AdvanceBio RP-Desalting column, and subsequently deconvoluted by way of Bioconfirm using a total entropy algorithm.

#### Negative-stain electron microscopy.

SEC-purified samples were diluted (0.005 mg ml^−1^ for oligomers and 0.05–0.1 mg ml^−1^ for crowns and cages) using SEC buffer immediately before application for 45 s to glow-discharged thick carbon film-coated 400 mesh copper grids (CF400-CU TH, Electron Microscopy Sciences). Grids were then stained and dried immediately twice using 2% uranyl formate. Dried grids were screened using a 120 kV Talos L120C transmission electron microscope. The E Pluribus Unum (EPU; FEI Thermo Scientific) software was used for automated data collection. Data processing was carried out in CryoSPARC (Structura Biotechnology).

An initial particle set of 100 particles was manually selected in cryo-SPARC10, followed by particle extraction and 2D classification. For dQS_T1, 345 micrographs were collected, which produced 56,692 particles, of which 9,870 particles (17.5%) were selected for 2D classification. For dQS_T1.5, 400 micrographs were collected, 64,684 particles were extracted and 10,938 particles (16.9%) were used for 2D classifications. For dQS_T3, 361 micrographs were collected, 22,237 particles were extracted and 1,784 particles (8.0%) were used for 2D classification. Finally, for dQS_T_hi, 353 micrographs were collected, and 2D classification was not performed due to the heterogeneity of the particles.

#### Cryo-EM data collection.

Three microlitres of purified dQS_T3 at 15 μM in 25 mM Tris-HCl and 300 mM NaCl (pH 8) was applied to glow-discharged 300 mesh R2/2 holey carbon C-flat grids. The grids were blotted using a Vitrobot Mark IV (Thermo Fisher Scientific) for 4–5 s at room temperature and at about 100% chamber humidity, then vitrified in liquid ethane. Frozen grids were stored in liquid nitrogen until screening and or data collection. The grids were initially screened for on-grid sample concentration and appropriate ice thickness for data collection using a Talos Arctica cryo-electron microscope (Thermo Fisher Scientific) operated at 200 kV. For data collection, dQS_T3 grids were imaged in a Titan Krios cryo-electron microscope (Thermo Fisher Scientific) operated at 300 kV using EPU data collection software (Thermo Fisher Scientific) and recorded on a K3 Summit electron detector with a GIF energy filter (Gatan) at a magnification of 81,000× corresponding to a calibrated sampling of 1.09 Å per pixel. Each image was composed of 40 individual frames with a total accumulated dose of 50 electrons per Å^2^. In total, around 5,100 movie stacks were recorded with a defocus range of −1 μm to −2.5 μm.

#### Cryo-EM image processing.

Micrographs were first motion-corrected using MotionCor2^31^ and the contrast transfer function of each motion-corrected image was determined using CTFFIND4^32^. An initial particle set of 100 particles was selected manually in cryoSPARC^33^, followed by particle extraction and 2D class averaging. Two-dimensional classes with recognizable particle features were subsequently used as input for Template Picking for the entire dataset of 3,346 micrographs, which produced 109,261 particles that were filtered to a dataset of 65,645 particles using Inspect Picks. The 65,645 particles were extracted at 1,024 pixels (px) and Fourier-cropped to 600 px for further analysis. In two rounds of 2D classification, 2D class averages with strong particle features were selected, and particles in 2D class averages with less-resolved features were removed. This resulted in a dataset of 40,365 particles that we used to generate three C1 ab-initio maps using cryoSPARC, in which one model had clear icosahedral symmetry with five-fold, three-fold and two-fold features. This map was used as a reference for further non-uniform 3D refinement (cryoSPARC) of the selected initial model class particle set of 24,393 particles with icosahedral symmetry imposed, yielding a 11.6 Å map (0.143 GSFSC criterion). We then re-extracted the refined particles to re-centre each particle using the refined orientations from the icosahedral refinement and symmetry-expanded the dataset with I symmetry, resulting in a set of 1,074,780 particles centred around each asymmetric unit. Using a focused mask centred around four trimers, an additional local refinement in cryoSPARC without imposed symmetry yielded a 7.1 Å map.

#### Model building and analysis.

For each refined map described above, rough atomic models were first generated of the respective assembly by docking the design model into the density using UCSF Chime-raX v.1.3 fit-in-map tool^33,34^. Each model was then flexibly fit into the respective density using ISOLDE^19^ Chime-raX plug-in until no further changes in the backbone positioning relaxed into the density were observed.

#### Cryo-ET data collection.

Three microlitres of purified dQS_T_hi at 15 μM in 25 mM Tris-HCl and 300 mM NaCl (pH 8) was applied to glow-discharged 300 mesh R2/2 holey carbon C-flat grids. The grids were blotted using a Vitrobot Mark IV (Thermo Fisher Scientific) for 4 s at room temperature and about 100% chamber humidity and vitrified by plunge freezing in liquid ethane. Frozen grids were stored in liquid nitrogen until data collection. For data collection, the grids were imaged on a Talos Arctica cryo-electron microscope (Thermo Fisher Scientific) operated at 200 kV under vacuum and liquid nitrogen temperature. Tilt series data were collected using SerialEM data collection software^35^ and images were recorded on a K3 Summit electron detector at a magnification of 22,000× corresponding to a calibrated sampling of 1.81 Å per pixel. Tilt series images were acquired using a bi-directional dose-symmetric collection scheme^36^ from negative 60° to positive 60° at 3° increments starting at 0°. Images were collected using low-dose settings with a dose rate of 3.33 e^−^ Å^−2^ s^−1^ at the sample, and a defocus range of −2 μm to −4 μm. Each tilt series was composed of 41 individual tilt images, with a total accumulated dose of 115 electrons per Å^2^. In total, 33 tilt series were collected.

#### Cryo-ET subvolume analysis.

Subvolume analysis steps were performed using EMAN2 v.2.3 Tomo pipeline^37^. A total of 1,296 tilt-image movie stacks with 6 frames per movie were motion-corrected using MotionCor2. The motion-corrected images for each tilt series were aligned and reconstructed into 3D tomogram volumes using e2_tomogram.py. The contrast transfer function estimation for each tilt image was performed using the EMAN program e2spt_tomoctf.py. Owing to the marked particle-to-particle heterogeneity, analysis focused on sub-particle regions that could be extracted from each particle subvolume. A total of 50 high-signal-to-noise-ratio sub-particle coordinates centred around visually identifiable pentamer assemblies were manually picked and extracted from tomograms with varying average defocus values. Subvolumes were extracted at box size 512 and binned by 4 to a box size of 128. The subvolumes were then used to generate an initial model using e2spt_sgd_new.py with C1 symmetry and five iterations. The same manual picking and initial model generation steps were performed for 50 high-signal-to-noise ratio sub-particles centred around hexamers. Each volume was then used for EMAN2 reference-based picking of sub-particles within all tomograms to generate sets of both pentamer and hexamer sub-particle regions.

For pentamers, the set of 1,416 template-picked and manually checked subvolumes binned by 4 was iteratively refined using e2spt_refine_new.py without imposed symmetry to 26.7 Å resolution (0.143 GSFSC criterion). The template-picked hexamer dataset, with 13,793 particles binned by 4, was similarly refined to 20.2 Å resolution. Refined coordinates and orientations of the refined penton subvolumes were mapped in 3D back to the originating tomogram using the EMAN2 program e2spt_mapptclstotomo.py.

Visualization, figure generation and model docking were performed using UCSF ChimeraX v.1.3 and its built-in tools.

## Extended Data

**Extended Data Table 1 |:** Designed sequence of dQS_T1, dQS_T1.5, dQS_T3 and dQS_T_hi with a HIS-tag at the C-term

ID	Protomer sequence
dQS_T1	GSPELFLQDLRSLVEAARILARLARQRGDEHALERAARWAEQAARQAERLARQARKEGNLELALKALQILVNAAYVLAEIARDRGNEELLEYAARLAEEAARQAIEIWAQAMEEGNQQLRTKAAHIILRAAEVLLEIARDRGNQELLEKAASLVDAVAALQQAAAAILEGDVLKAFEAAREALKAAEKAGDKDMLKAVAIAVARIIEEAAANDADVEALEKAAEVAREALEAAIKYGLYEEAKKVVIEVAKLAVKAKNQDVIAKALEVALKAAEKLIKKGVFEEAKEILEELVKALEKLKNLDQDALAKAADAAYRIAHEALKQGNLEVALAASKVAMAALKQTGGSGGSHHHHHH
dQS_T1.5	GSPELFLQDLRSLVEAARILARLARQRGDEHALERAARWAEQAARQAERLARQARKEGNLELALKALQILVNAAYVLAEIARDRGNEELLEYAARLAEEAARQAIEIWAQAMEEGNQQLRTKAAHIILRAAEVLLEIARDRGNQELLEKAASLVDAVAALQQAAAAILEGDVRKAVEAAEEAVRAAEEAGDIDMLRAAVIAAARVGARAAELGAEPEVLRRAVEVILRALRAAIERGDIDLAVLALRALARLIEEITQPEAVDAAILAALEALLEAAELKDPAVEVAVVVVLEAATKKMHLATQDALAKIADLAYRLAHEYLKIGNLAVALAASKLAMAALNQTGGSGGSHHHHHH
dQS_T3	GSPELFLQDLRSLVEAARILARLARQRGDEHALERAARWAEQAARQAERLARQARKEGNLELALKALQILVNAAYVLAEIARDRGNEELLEYAARLAEEAARQAIEIWAQAMEEGNQQLRTKAAHIILRAAEVLLEIARDRGNQELLEKAASLVDAVAALQQAAAAILEGDVVKAAEAAKEAVKAAAEAGDEDMLRAAAIAVERIAKKAAEIVTDNEEAEKLVEILVEILKAVAEAGDEDAALKIAKAVLIAAKVTTNNEAVEKALEAAVEAAEKFVERGDEKAALEFAKAALELLKLLKPESQDALAKVADRAYRLAHEALKRGNLKVALAFSKVAMAALEQTGGSGGSHHHHHH
dQS_T_hi	GSPELFLQDLRSLVEAARILARLARQRGDEHALERAARWAEQAARQAEKLARQARKEGNLELALKALQILVNAAYVLAEIARDRGNEELLEYAARLAEEAARQAAEIWAEAARRGNQQLRTKAAHILLRAAEVLLEIARDRGNQELLEKAQRIVEAVAAAQQVAALALRLAEELDSEEAKKAVRAIAEAAAAALLAALQGKDEVAKLALKVLKEAIELAKENRSEEALKVVLEIARAAAAAARAAEEGKTEVAKLALKVLEEAIELAKENRSEEALKVVLEIARAALAAAQAAEEGKTEVAKLALKVLEEAIELAKENRSEEALKVVLEIARAALAAAQAAEEGKTFIAKAALRILEEAIEMAKENRSEEALKQVLEIARAAYDAAEAAREGKGGSGGSHHHHHH
dQS_hex	GSPELFLQDLRSLVEAARILARLARQRGDEHALERAARWAEQAARQAERLARQARKEGNLELALKALQILVNAAYVLAEIARDRGNEELLEYAARLAEEAARQAIEIWAQAMEEGNQQLRTKAAHIILRAAEVLLEIARDRGNQELLEKAASLVDAVAALQQAAAAILEGDVKKAAEAAAEAARAAAEAGDLDMLRAVGIAAERIAKEAKKAGDEKEEGLEIFEEIARLLVEAVEKGDKDLAKMLARGLELAAQLLAAESQDELAKIADQAYRAAHEALKEGNLDAALLASKVAMEALKRTGGSGGSHHHHHH

**Extended Data Table 2 |:** CryoEM data collection and map processing statistics for dQS_T3

Data Collection		
Microscope	Titan Krios	
Voltage (kV)	300	
Detector	Gatan K3	
Energy Filter	Gatan BioQuantum Gif	
Recording mode	Counting	
Magnification	81,000 X	
Movie micrograph pixel size (Å)	1.09	
Dose rate (e^−^/Å^2^/s)	9.66	
No. of frames per movie micrograph	40	
Frame exposure time (s)	0.05	
Movie micrograph exposure time (s)	2	
Total dose (e^−^/Å^2^)	50	
Under focus range (μm)	0.8–2	
Number of movie micrographs	3,346	
Map Processing		
	dQS_T3 particle EMDB ID: EMD-70787	Focused pentamer region EMDB ID: EMD-70792
Extraction Box Size (pix)	1200 (fourier cropped 720)	1200 (fourier cropped 720)
Initial particle images (no.)	65,645	1,074,780
Final particle images (no.)	24,393	1,074,780
Symmetry imposed	I	C1
Map resolution (Å)	11.3	7.1
FSC threshold	0.143	0.143
**Refinement**	N/A	

**Extended Data Table 3 |:** CryoEM data collection and map processing statistics for dQS_T_hi

Data Collection		
Microscope	Glacios	
Voltage (kV)	200	
Detector	Gatan K3	
Energy Filter	N/A	
Recording mode	Counting	
Magnification	22,000X	
Movie micrograph pixel size (Å)	1.81	
Tilt scheme	Dose symmetric	
Starting tilt angle	0°	
Tilt series tilt increment	3°	
Tomogram tilt range	−60° to 60°	
Number tilt images per tilt series	41	
Dose rate (e^−^/Å^2^/s)	3.33	
No. of frames per movie micrograph	6	
Frame exposure time (s)	0.14	
Tilt movie micrograph exposure time (s)	0.839	
Total dose (e^−^/Å^2^)	115	
Under focus range (μm)	2–4	
Number of movie micrographs	1296	
Number of tilt series	33	
Map Processing		
	Focused pentamer region EMDB ID: EMD-70797	Focused hexamer region EMDB ID: EMD-70798
Extraction Box Size (pix)	512 (4x binned to 128)	512 (4x binned to 128)
Initial particle images (no.)	1416	13,793
Final particle images (no.)	1274	13,103
Symmetry Imposed	C1	C1
Map resolution (Å)	26.7	20.2
FSC threshold	0.143	0.143

## Supplementary Material

Supporting_information

Sample_Code

**Supplementary information** The online version contains supplementary material available at https://doi.org/10.1038/s41586-026-10554-z.

## Figures and Tables

**Fig. 1 |:**
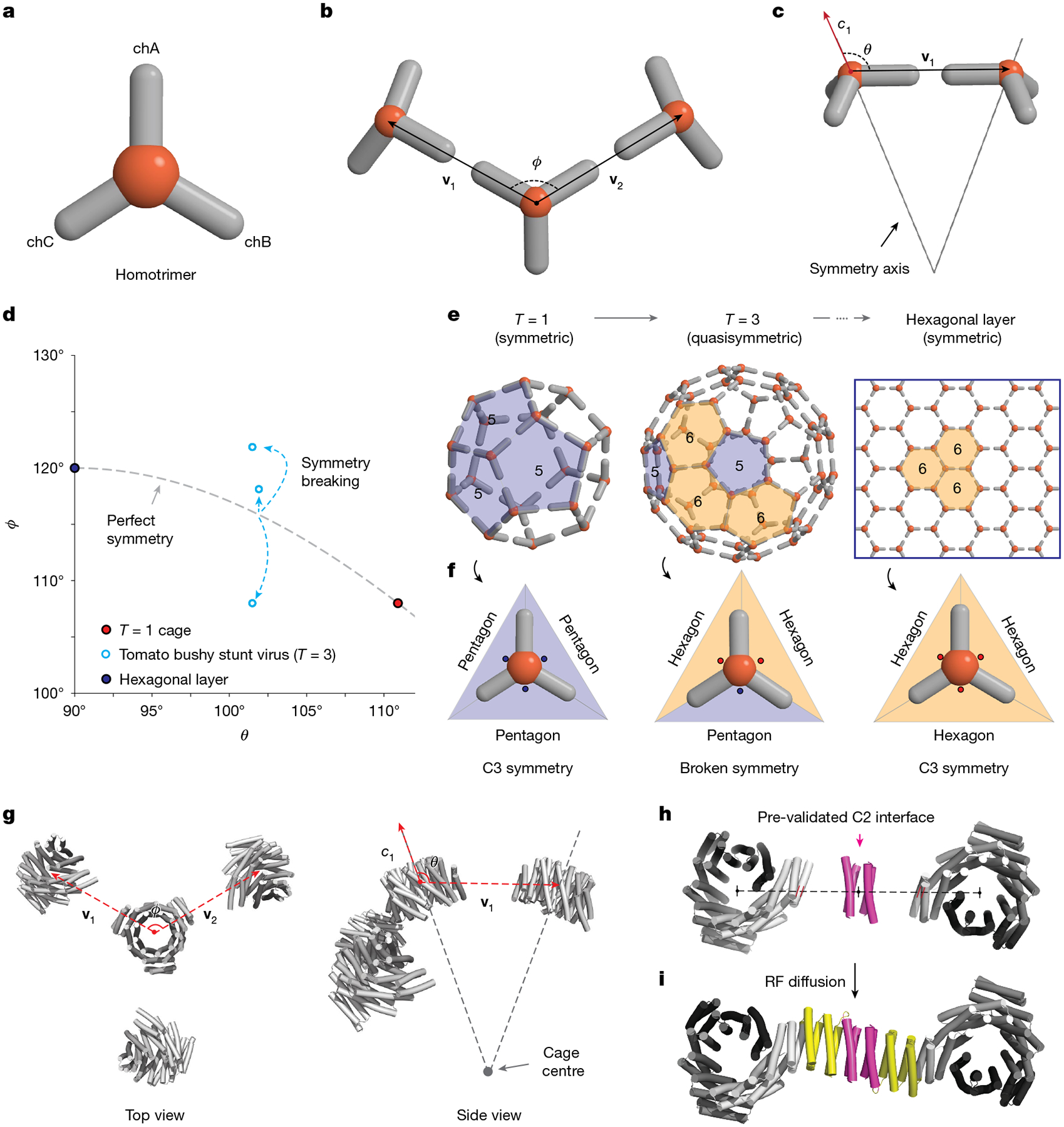
Quasisymmetry design parameter space. **a**, Schematic of a homotrimeric building block model consisting of three identical chains (chA, chB and chC) shown with grey arms extending from an orange central hub. **b**, *ϕ* is defined as the angle between the vectors **v**_1_ and **v**_2_ from the COM of a trimer to those of two nearest neighbours. **c**, *θ* is defined as the angle between the C3 symmetry axes (*c*_1_) of adjacent trimers. **d**, Parameter space defined by *θ* and *ϕ*. The grey dashed line represents the correlation between *θ* and *ϕ* for perfect C3 symmetry. **e**,**f**, Schematic of symmetry breaking in quasisymmetric cages. The local environment of trimeric building blocks around the *T* = 1 cage (**f**, left) and the 2D hexagonal lattice (**f**, right) is C3-symmetric with three identical pentagons or hexagons. By contrast, the local symmetry of the *T* = 3 cage is broken (**f**, middle) by the different geometries of the pentons and hexons. **g**–**i**, Computational protocol for designing quasisymmetric cages for given *θ* and *ϕ*. Homotrimers are placed for given *θ* and *ϕ* (**g**). A homodimeric C2 interface is placed at the centre of two homotrimers (**h**). RFdiffusion generates a new protein backbone that rigidly connects the C2 and C3 protomers (**i**).

## Data Availability

Cryo-EM maps have been deposited in the Electron Microscopy Data Bank (accession codes EMD-70787, EMD-70792, EMD-70797 and EMD-70798). All other data are available in the paper or the Supplementary Information.

## Code availability

The source code for RFdiffusion backbone generation is freely available at GitHub (https://github.com/RosettaCommons/RFdiffusion). The source code of ProteinMPNN for the sequence design is freely available at GitHub (https://github.com/dauparas/ProteinMPNN).
