# Supplementary material for: Design of one-component quasisymmetric protein nanocages
Source: Nature. Author manuscript; Available in PMC 2026 Aug 5. (PMC13437138; doi:10.1038/s41586-026-10554-z)
Supplement: Supplementary file 1 [file NIHMS2197401-supplement-Supporting_information.pdf]

nature portfolio

https://doi.org/10.1038/s41586-026-10554-z

Supplementary information

Design of one-component quasisymmetric protein nanocages

In the format provided by the authors and unedited

Supplementary Figures

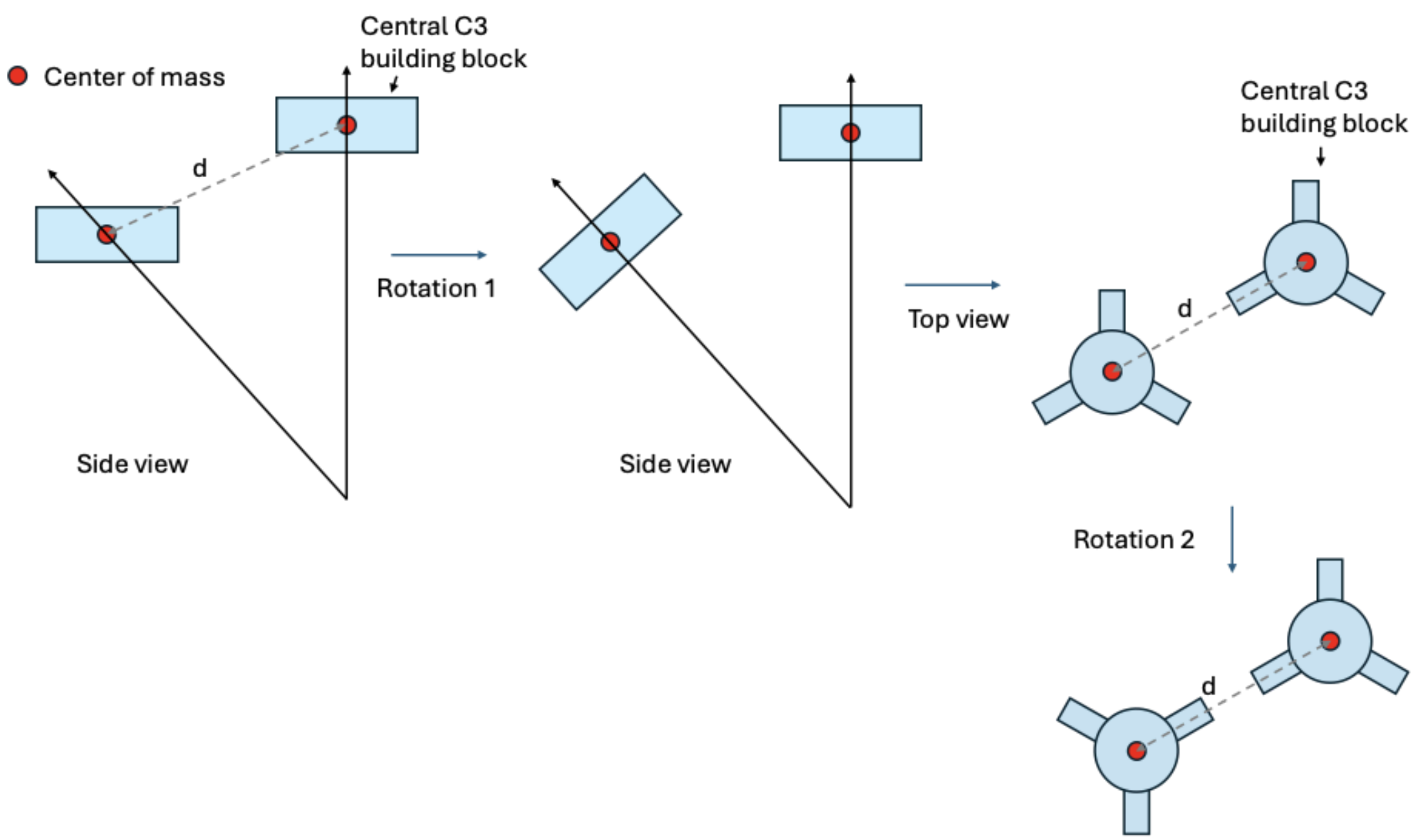

Figure S1. Rotation 1 and 2. Schematics of two rotations of C3 building blocks for a given set of parameters (θ and ϕ), which is used for generating input configurations of RFdiffusion (see *Model construction* part in the computational method section).

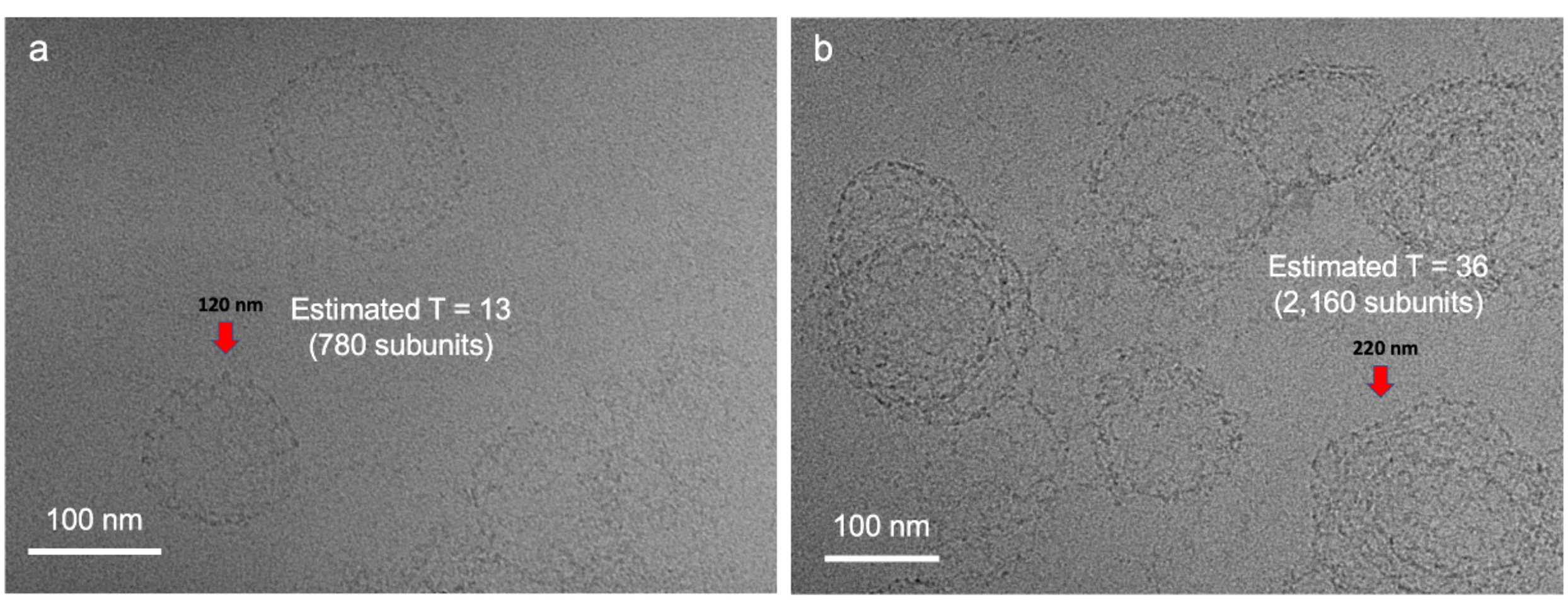

Figure S2. Cryo-EM micrographs of dQS_T_hi. The diameters of cages found in dQS_T_hi are diverse and ranging from (a) 120nm - (b) 220nm.

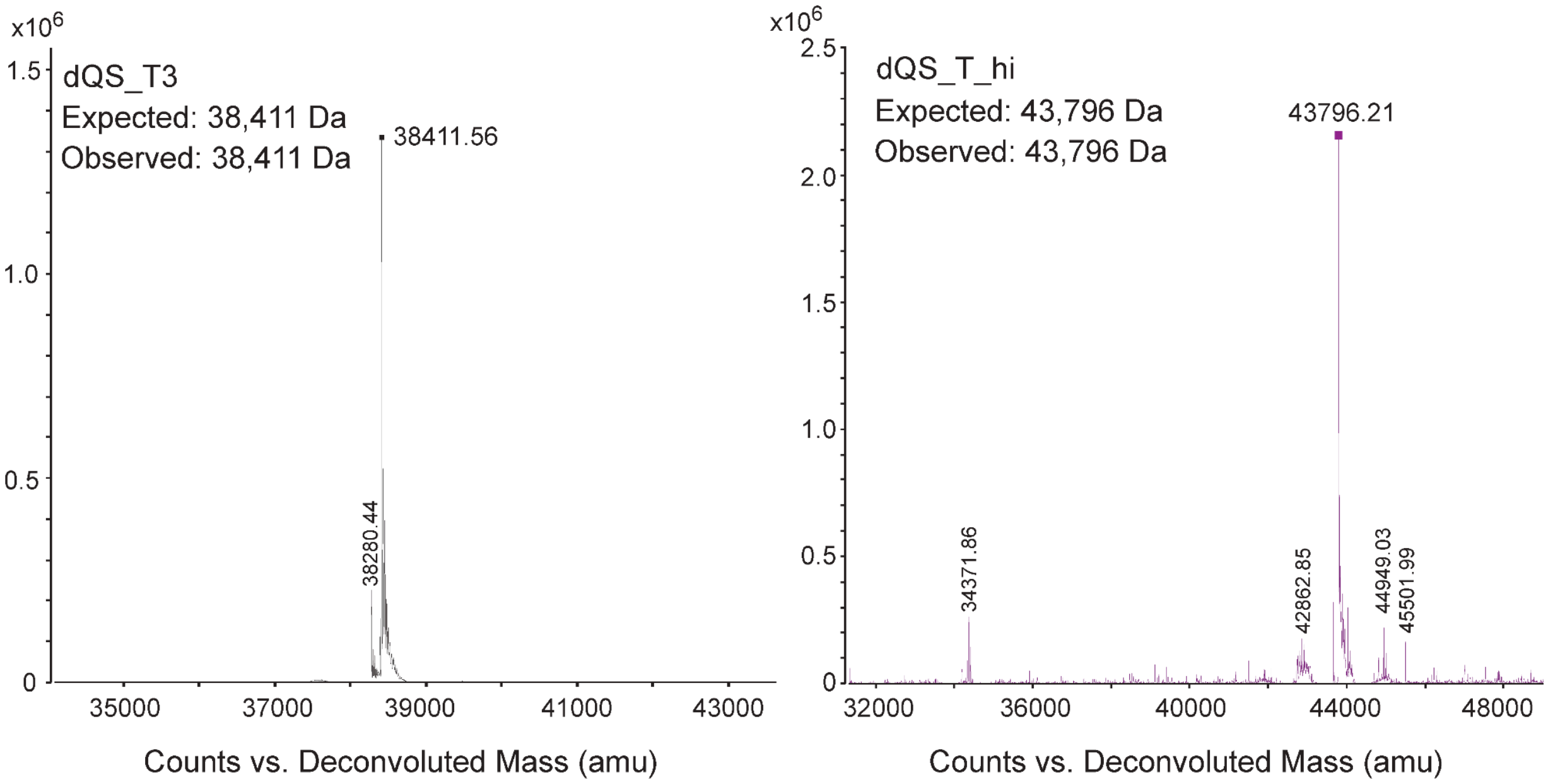

Figure S3. LC-MS results of (left) dQS_T3 and (right) dQS_T_hi show a peak at the expected mass of the designed protein.

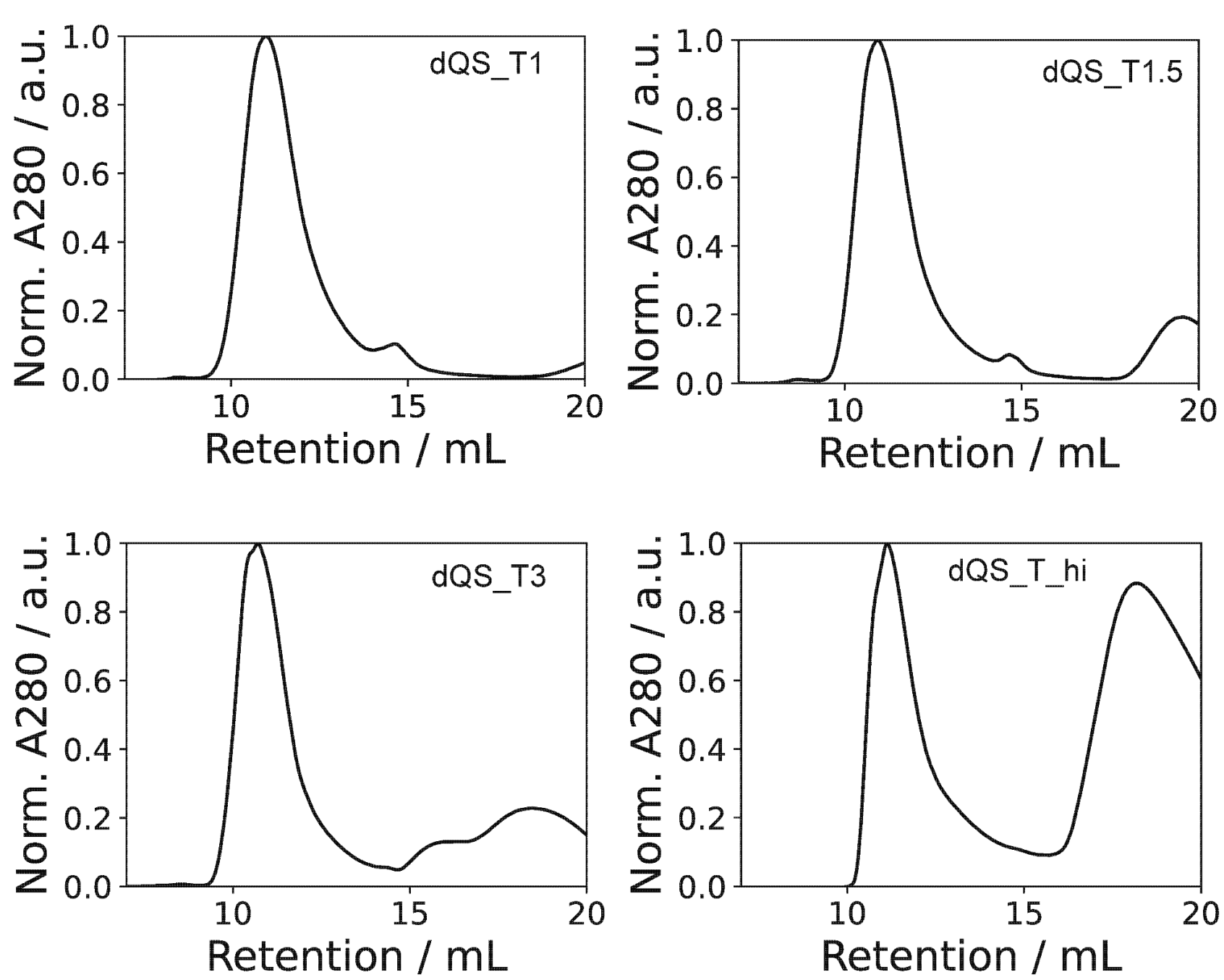

Figure S4. SEC traces of dQS_T1, dQS_T1.5, dQS_T3 and dQS_T_hi obtained using Superdex 6 10/300 increase column. Peak around ~10ml retention volume indicates the cage peak.

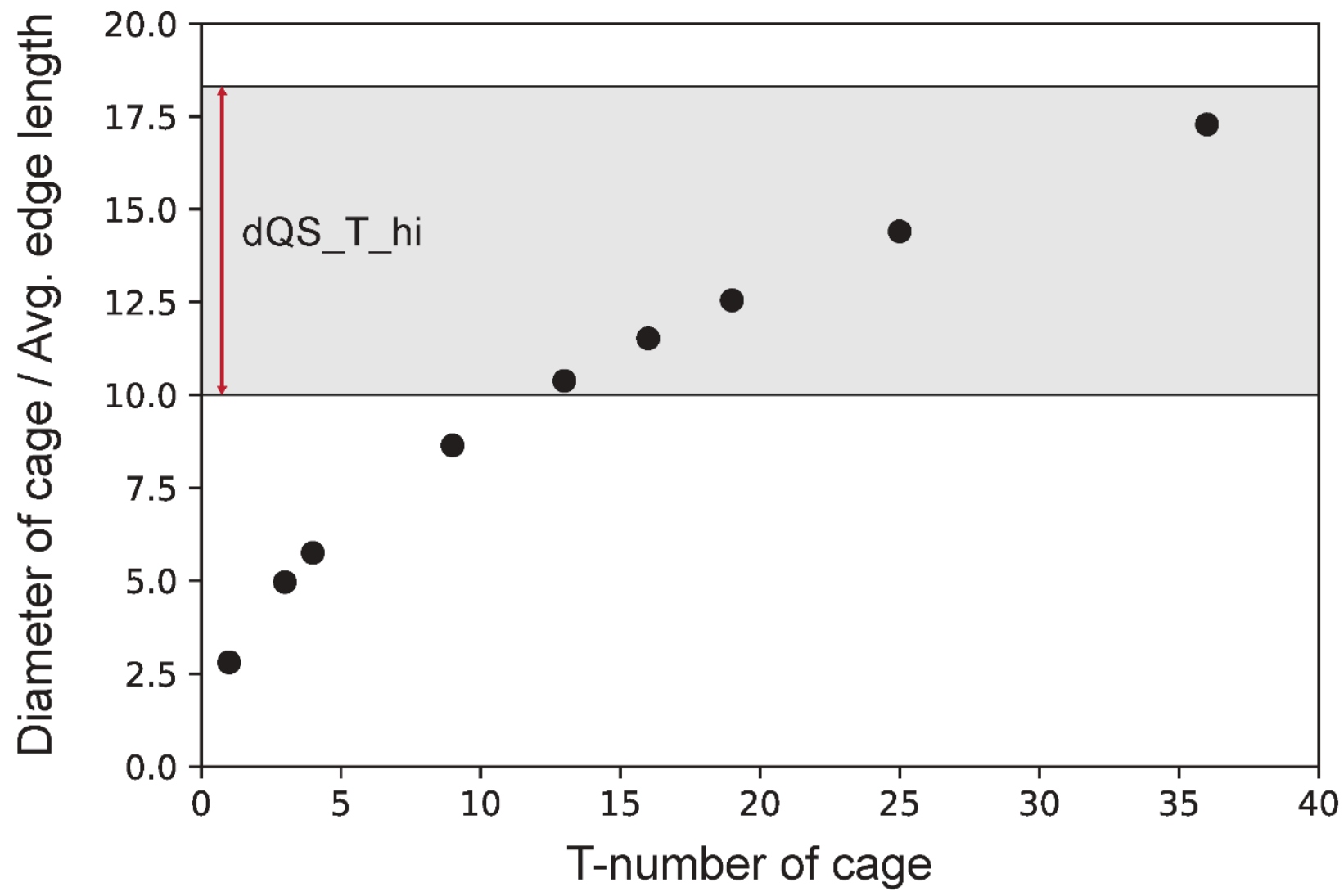

Figure S5. The relationship between T-number and the ratio between the edge length and the diameter of the cages. The diameter of cages observed in dQS_T_hi are ranging from 120nm to 220nm, and the edge length is ~12nm, which suggests that the T-number of observed cages are ranging from $13 \leq T \leq 36$.

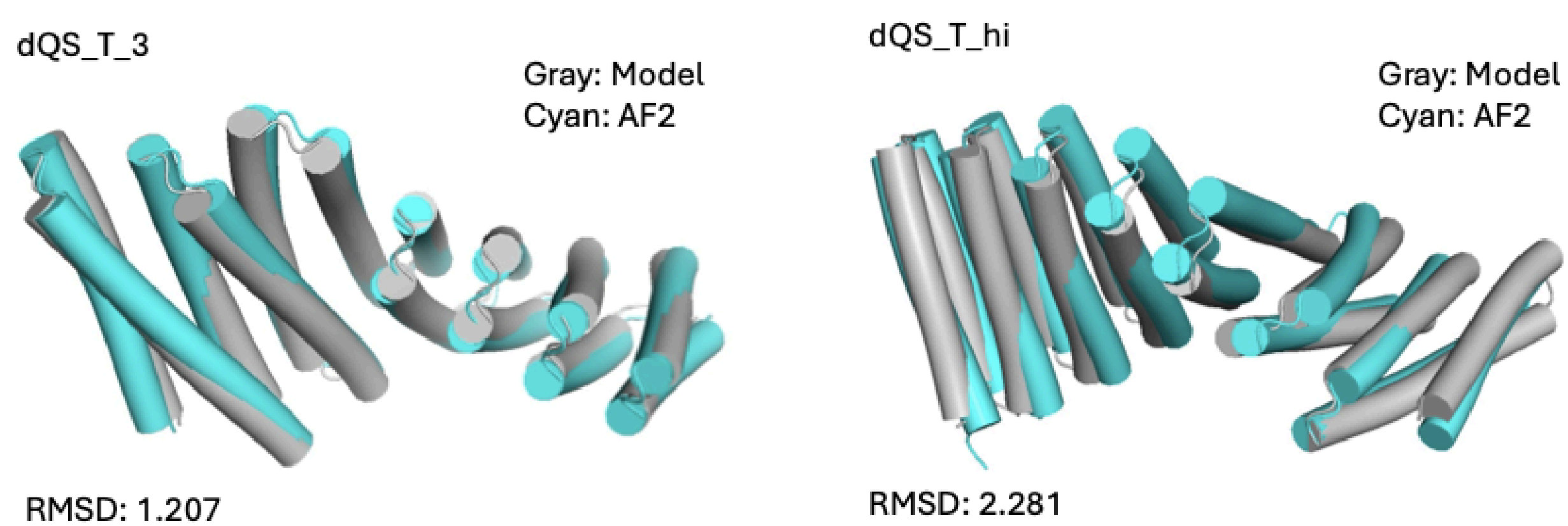

Figure S6. Alignment of AlphaFold-2 predictions to the model structure of dQS_T3 and dQS_T_hi monomers.

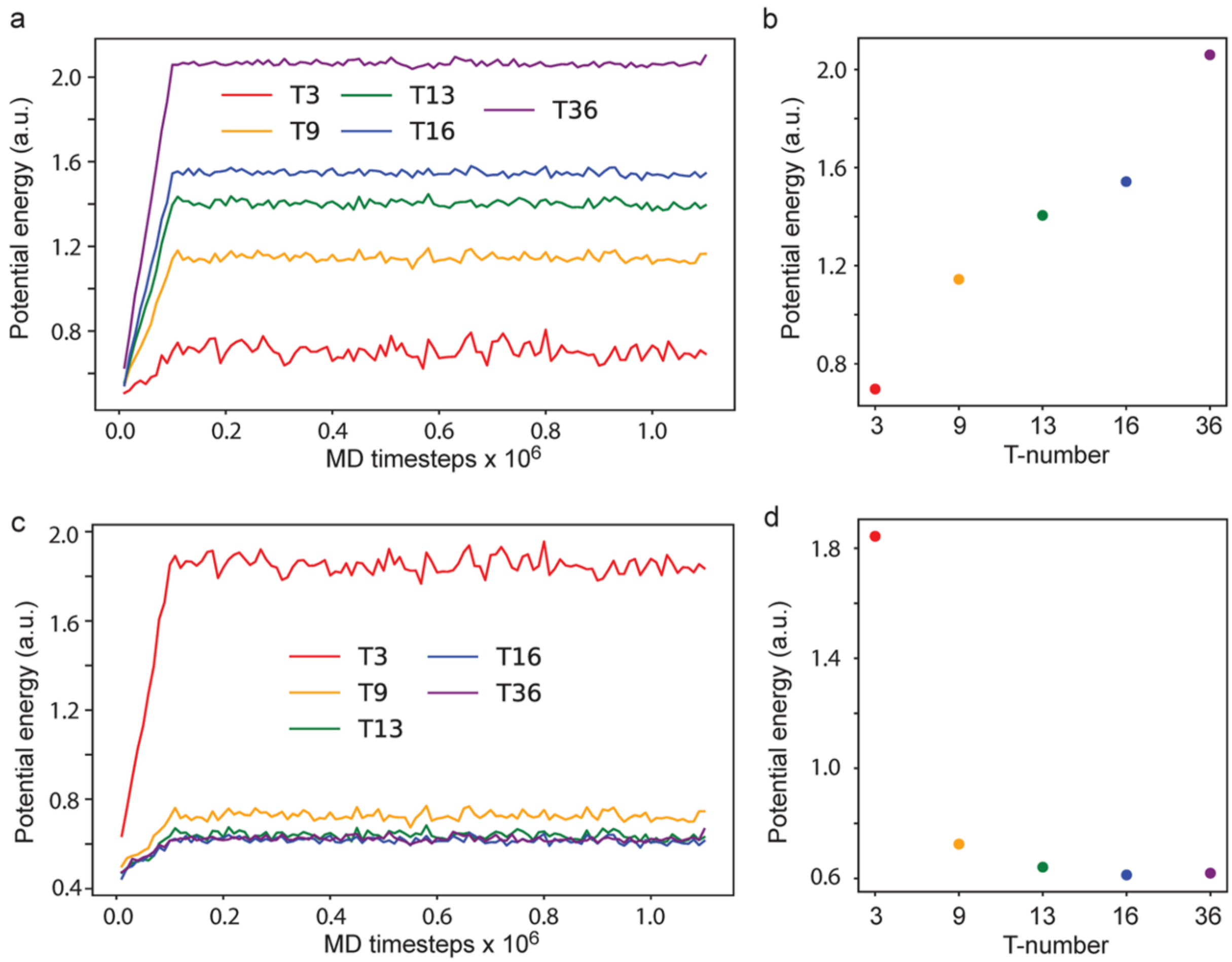

Figure S7. MD simulations for the potential energy calculation across the diverse T-number cages. (**a,c**) Potential energy calculation for T=3, 9, 13, 16 and 36 with a set of potential energy parameters optimized for (**a**) the T=3 and (**c**) T=13. (**b,d**) Averaged potential energy of each T-number for 10^5 MD timesteps in equilibrium with a set of potential energy parameters optimized for (**b**) the T=3 and (**d**) T=13.

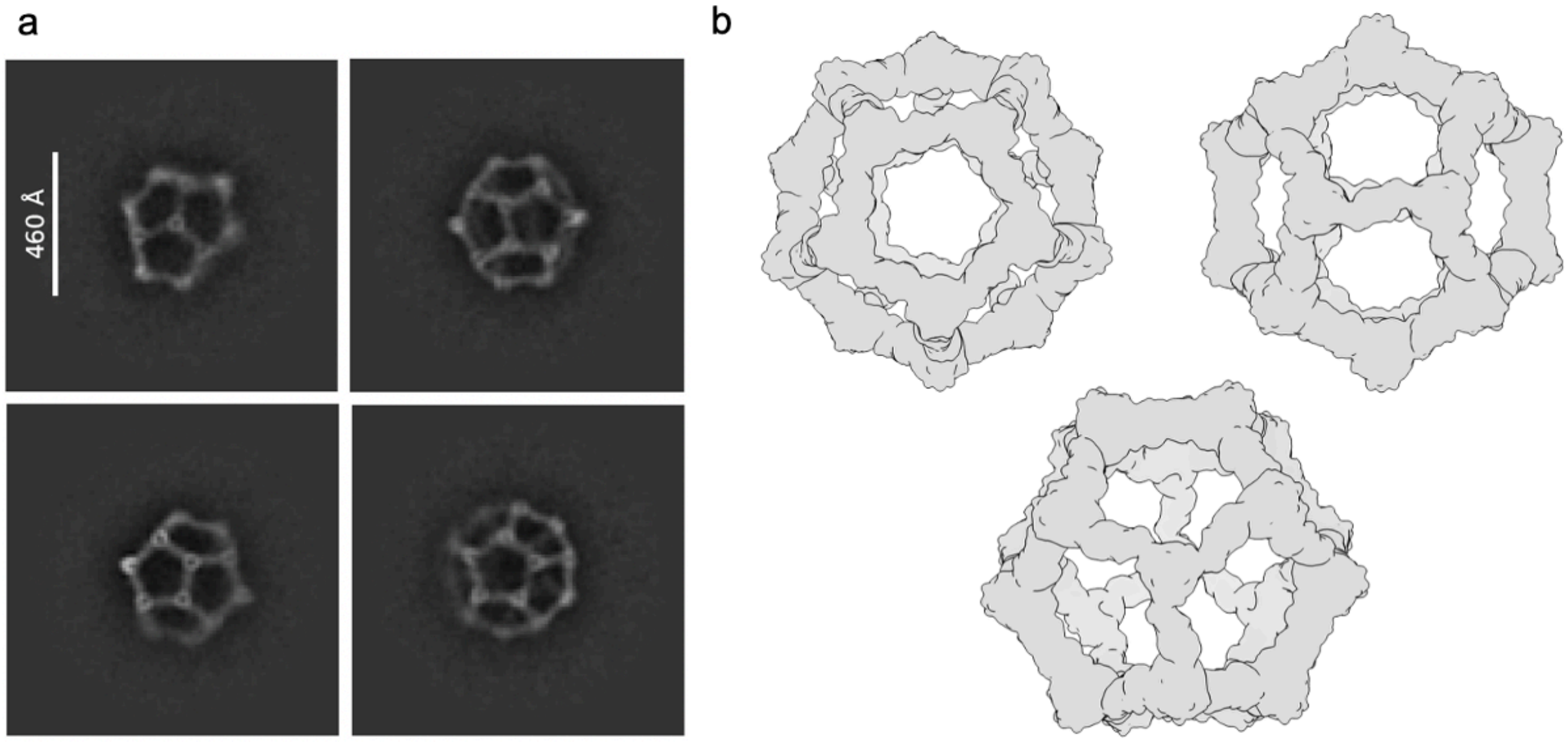

Figure S8. nsEM 2D classifications and 3D reconstruction of dQS_T1. (**a**) Four representative 2D classification averages. (**b**) 3D reconstruction map viewed from three different rotational symmetry axes (5-fold, 2-fold, 3-fold).

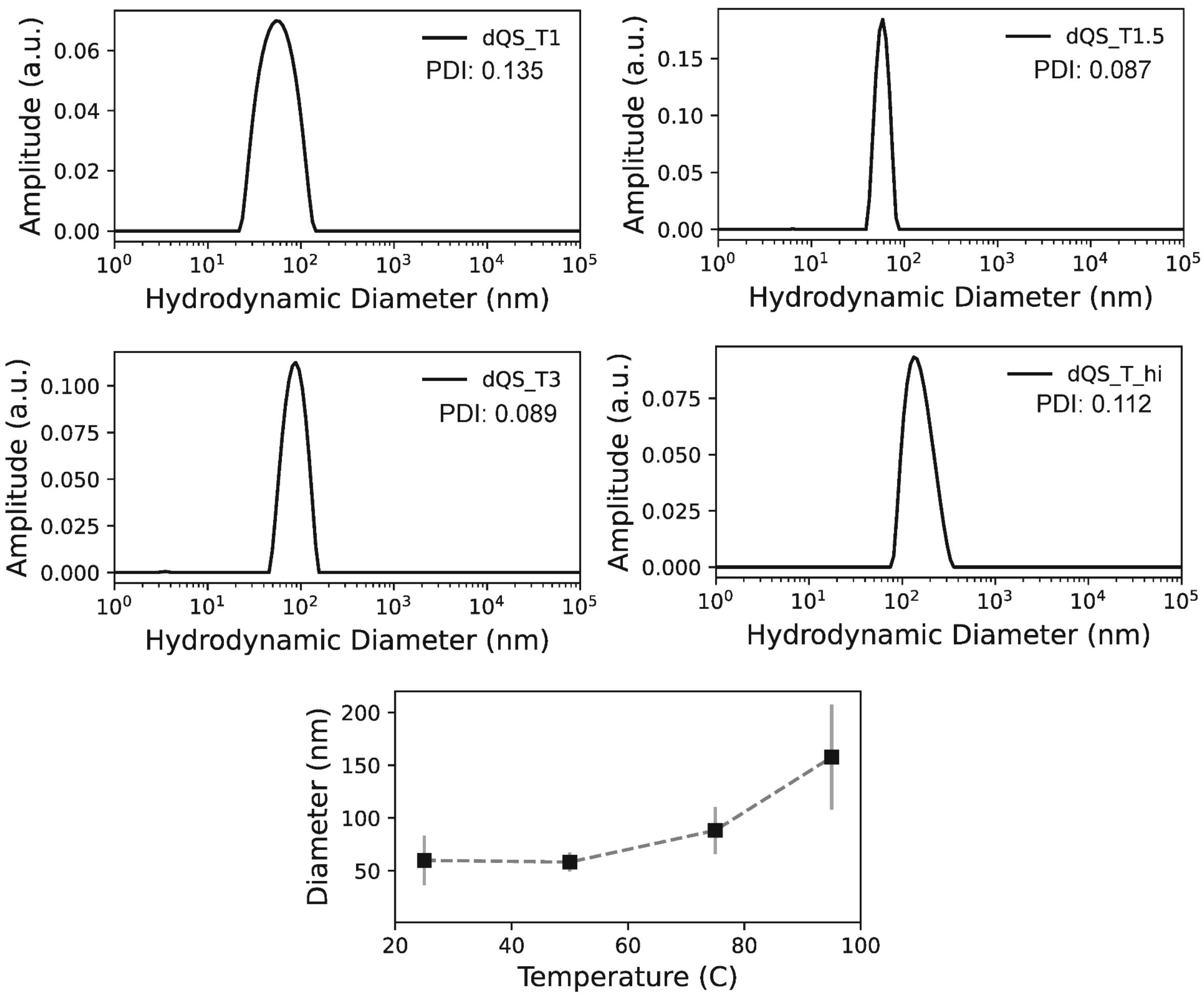

Figure S9. Dynamic light scattering (DLS) analysis of dQS_T1, dQS_T1.5, dQS_T3, and dQS_T_hi. Summary of the four systems is shown at the bottom. The error bars in the summary data indicate the standard deviations of the distribution of hydrodynamic diameter obtained from DLS.

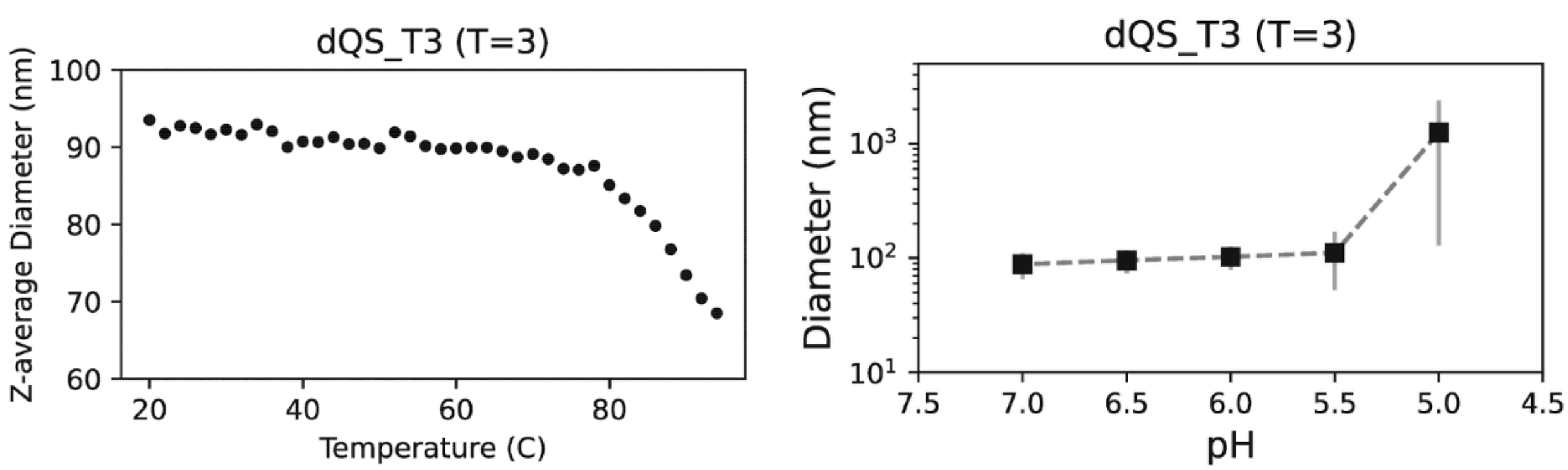

Figure S10. Stability analysis of dQS_T3 for different (left) temperatures and (right) pHs using DLS. The dQS_T3 starts to be disassembled over 75°C and aggregated between pH = 5.5 and 6.0. The error bars in the right figure indicate the standard deviations of the distribution of hydrodynamic diameter obtained from DLS.

Sample code descriptions

We have provided a sample code as a supplementary file for constructing model structures for a given set of parameters (d, θ and ϕ). The outputs of the sample code are used as an input of the RFdiffusion. Detailed descriptions of the model construction idea can be found in the Method section.
